# Advances in the design, generation, and application of tissue-engineered myocardial equivalents

**DOI:** 10.3389/fbioe.2023.1247572

**Published:** 2023-09-22

**Authors:** Giacomo Bernava, Laura Iop

**Affiliations:** Department of Cardiac Thoracic Vascular Sciences and Public Health, Padua Medical School, University of Padua, Padua, Italy

**Keywords:** cardiovascular diseases, cardiac tissue engineering, myocardium, disease modeling, regenerative medicine

## Abstract

Due to the limited regenerative ability of cardiomyocytes, the disabling irreversible condition of myocardial failure can only be treated with conservative and temporary therapeutic approaches, not able to repair the damage directly, or with organ transplantation. Among the regenerative strategies, intramyocardial cell injection or intravascular cell infusion should attenuate damage to the myocardium and reduce the risk of heart failure. However, these cell delivery-based therapies suffer from significant drawbacks and have a low success rate. Indeed, cardiac tissue engineering efforts are directed to repair, replace, and regenerate native myocardial tissue function. In a regenerative strategy, biomaterials and biomimetic stimuli play a key role in promoting cell adhesion, proliferation, differentiation, and neo-tissue formation. Thus, appropriate biochemical and biophysical cues should be combined with scaffolds emulating extracellular matrix in order to support cell growth and prompt favorable cardiac microenvironment and tissue regeneration. In this review, we provide an overview of recent developments that occurred in the biomimetic design and fabrication of cardiac scaffolds and patches. Furthermore, we sift *in vitro* and *in situ* strategies in several preclinical and clinical applications. Finally, we evaluate the possible use of bioengineered cardiac tissue equivalents as *in vitro* models for disease studies and drug tests.

## 1 Introduction

Heart failure is the fatal epilogue of many cardiovascular diseases that impair ventricular filling or blood ejection. The number of affected patients is dramatically increasing worldwide with a median annual incidence of 3.2 cases per 1,000 people in the European countries (Seferović et al., 2021) raising to 27 cases for every 1,000 Medicare beneficiaries in the US (Heidenreich et al., 2022). These epidemiologic data are expected to vary following the increasing aging population but also in function to the adoption of a recent, universal definition of heart failure (Bozkurt et al., 2021) and to advanced prediction methods, such as machine learning, able to sensibly improve diagnosis and risk stratification (Uijl et al., 2021; Nakano et al., 2023; Takahama et al., 2023).

Among the heterogeneous pathologies causing heart failure, ischemic heart disease and myocardial infarction are responsible for particularly high age-adjusted mortality rates, especially in the Caribbean and Central America, the Balkans, the Persian Gulf, Southeast Asia, as well as in West Africa, Eastern Mediterranean, and Northern Europe (Tsao et al., 2023). In the presence of a stenosis or the case of a different, non-obstructive substrate, the hemodynamic dysfunction of an artery occurring in acute coronary syndrome induces an ischemic condition for the cardiomyocytes residing in the perfused area. Depending on the entity and persistence of this ischemic insult, the damage to these cells might be irreparable and trigger a cascade of events ultimately ending in non-contractile, fibrotic scar tissue.

Clinical treatments available so far aim to promptly restore blood perfusion through percutaneous coronary intervention or coronary artery bypass, also depending on the number of interested vessels, as well as to reduce inflammation by means of pharmacological therapies that should prevent any further aggravation towards heart failure.

Other therapeutic approaches have been attempted in the last 20 years following regenerating strategies aiming at restoring the physiologic contractility in scarred regions. After the paradigm reversal of the heart as a fully differentiated organ (TAM et al., 1995), cardiac stem cell niche stimulation was pursued through the infusion of growth factors and/or cells. The encouraging outcomes observed in animal models of cardiac ischemia prompted the clinical application, however, with effects generally inadequate for an effective improvement of heart function (Clifford et al., 2012; Fisher et al., 2016; Menasché, 2018). Per se, cells or growth factors alone might difficultly reconstruct the damaged myocardial tissue due to a regeneration-adverse, cytotoxic microenvironment imposed consequently to the injury. Therefore, although some strategies are still pointing to improving cell commitment through paracrine signaling [for example (Yin and Jiang, 2023)], a tissue replacing approach is currently under main consideration with the rationale of substituting the scar tissue with a physiologic myocardial equivalent able to integrate with surrounding healthy regions and re-establish the heart pump work. In such a perspective, the goal of cardiac tissue engineering is the opportune combination of different elements in order to generate a functional, bioengineered myocardial equivalent. The generation of complete cardiac tissue is not a recent enterprise, and the first attempts parallel the initial experiences with cellular and/or cytokine infusions. Some of the developed technologies began to be applied in clinics with promising results [as an example (Yamamoto et al., 2019)].

This review intends to offer a panoramic overview on cardiac engineered tissues manufactured so far, by exploring the concepts and efforts faced during their development. A particular emphasis will be given to the challenging task of increasing biomimetics towards the native extracellular matrix, while achieving and sustaining cardiac tissue maturation and functionality in preclinical and clinical applications for cardiac regeneration. Furthermore, more recent propositions of cardiac tissue engineering will be inspected in the field of disease modeling and pharmacological testing.

## 2 Biomimetic design: from extracellular matrix to scaffold

### 2.1 The relevant role of the extracellular matrix in cellular, tissue, and organ function

In all native tissues, cell behaviors are influenced by the surrounding dynamic microenvironment, the extracellular matrix (ECM), which transmits instructions and information through biochemical and biophysical signaling. However, ECM also plays the crucial role of providing structural support to cells. Indeed, the composition and local distribution of ECM components dictate their 3D architecture, depending on proteins’ type and concentration (Wang et al., 2018). Proteoglycans, collagens, elastin, and fibronectin provide structural support to the cells and give specific mechanical properties to the tissue, while growth factors, cytokines, chemokines, and other bioactive molecules are anchored to the ECM network in order to provide biochemical stimuli and thus acting as a reservoir (Frantz et al., 2010). Arrangement of matrix proteins with GAGs and glycoproteins determines the geometry, topography, porosity, density, and mechanical stiffness of the ECM, which further regulates many cellular fate processes. In addition, micro/nanoscale topography created by organized fibrillar bundles appears to have a profound impact on cell shape, cytoskeletal structure organization, and intercellular signaling (McBeath et al., 2004; Nelson et al., 2005).

ECM can cover different functions in tissue physiology. Two specific categories of ECM can be defined: basement membrane and interstitial matrix. The first is a thin, compact layer - composed mainly by IV collagen, laminins, and proteoglycans - with the role to separate tissues within the body, usually in contact with epithelium and endothelium (Paulson, 1992). The second includes all other ECM between cells in tissues and is composed by complex proteoglycans, in form of hydrated gel, glycosaminoglycans, as hyaluronan, and fibrous proteins, in particular type I collagen (Frantz et al., 2010). The basal membrane provides structural support in tissues and is essential in cell behavior, including cell adhesion, migration, and compartmentalization. Conversely, the interstitial membrane participates in signaling as much as it does in structure formation, respecting proposed model of binding-mediated hindered diffusion (Valdoz et al., 2021). Furthermore, the essential role to anchor cells at the ECM is covered by integrin, selectins and cadherins cell receptors. Indeed, integrins are essential in adhesion structure and transduce external stimuli inside the cell. They are heterodimer proteins composed by α and β glycoprotein subunits, each consisting of an extracellular portion terminating with a globular shaped head, a multidomain “leg”, a transmembrane helix and a cytoplasmatic tail region. Integrins generally are ECM-specific protein components, which possess an RGD (consisting in Arg-Gly-Asp triplet) integrin-binding domain, whose location and accessibility play a pivotal role in protein binding. The most common substrates for human integrins are laminins, collagen, and fibronectin, each of which possesses specific and characteristic α/β subunits (Moreno-Layseca et al., 2019). Depending on the composition of these ECM proteins, integrins initiate a signaling cascade to regulate cell proliferation, survival, and migration (Schussler et al., 2022). Eventually, different tissues can exhibit various degrees of stiffness, and ECM composition depends directly on tissue-specific mechanical properties and features (Wang et al., 2018). In fact, the ECM is organized in hierarchical structures consisting of micro- and nanoscale topographic patterns that are essential to the tissue for its exclusive function and mechanical properties. Matrix network composition, for example, collagen and/or elastin bundles, or external tissue forces, for example, contraction, bone mechanical loading or blood flow, could give specific orientation stimuli to the cells, changing their shape and cytoskeleton organization (Guimarães et al., 2020). All these mechanical, physical, and topographical stimulations activate the mechanosensing pathway. By mechanotransduction systems, cells translate these stimuli into biochemical signals controlling multiple aspects of cell behavior, including growth, differentiation, and migration. In this molecular process, integrins play an important role because they are also mechanotransductors, namely, they respond to mechanical forces with biochemical stimuli. Kinase cascades transduce signals at the cytosolic level, which activate some downstream effectors, as transcriptional co-activators factors YAP (Yes-associated protein) and TAZ (transcriptional coactivator with PDZ-binding motif, also known as WWTR1). When activated, the latter translocate into the nucleus, leading to the transcription of specific gene targets (cell cycling, survival, cell fate regulators, *etc.*) (Dupont et al., 2011).

### 2.2 The elementary bricks of the heart

**FIGURE 1:**
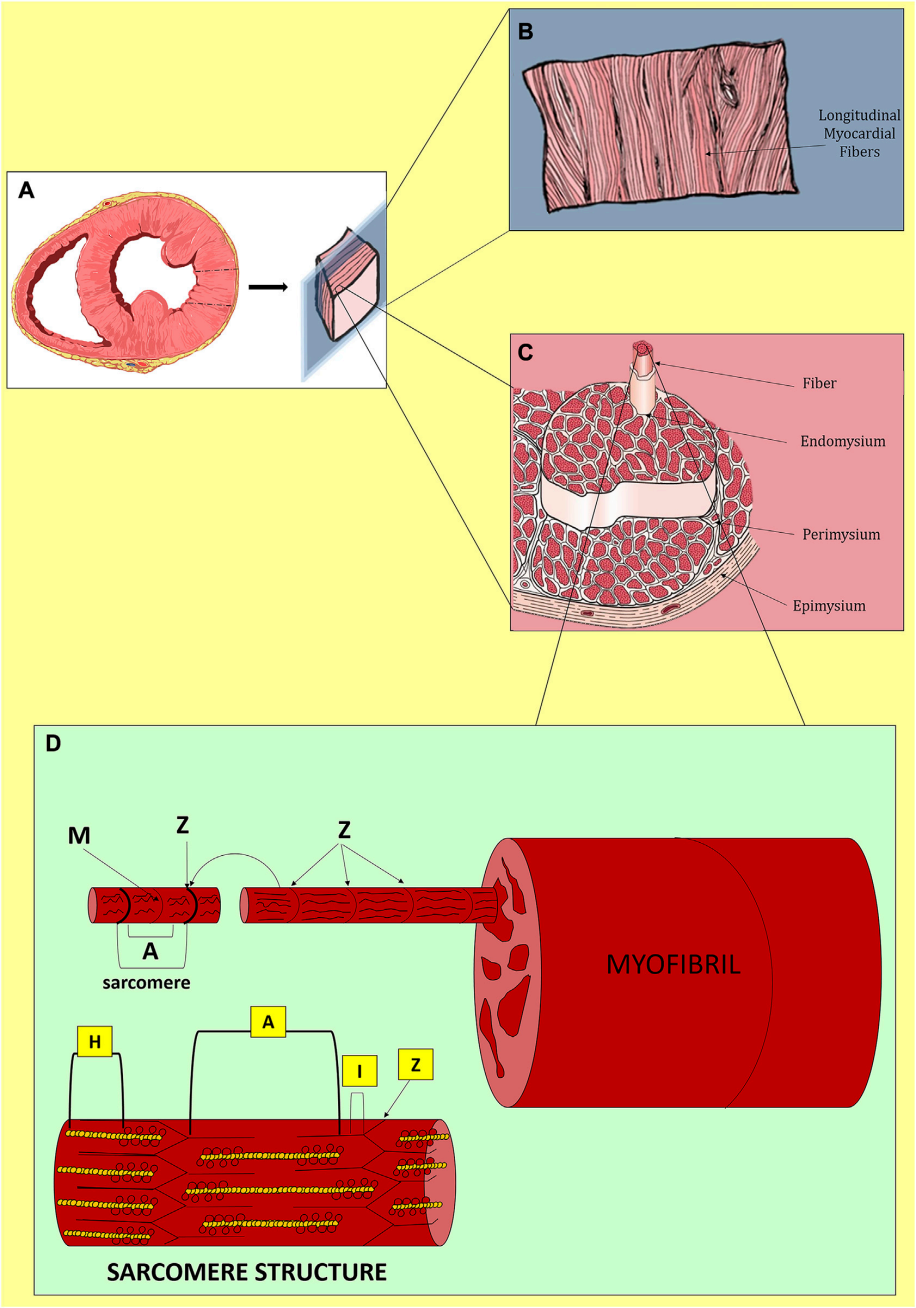
Native myocardium in physiological conditions. **(A)** Transversal section of the cardiac ventricles. **(B)** Longitudinal distribution of myocardial fibers. **(C)** Organization of myocardial fibers. **(D)** Structure of a sarcomere with Z-discs at both extremities, longitudinally distributed A-bands composed of myosin and actin, I-bands rich in actin and titin and disposed between Z-discs and A-bands, in the middle, H-zones with only myosin and cross-connected centrally by M-lines. During contraction, the filaments of myosin and actin slide.

Heart function is the orchestration of electrical and mechanical periodicity in a contractile machinery mainly composed of cardiac myocytes (CMs) and ECM (Figure 1). The heart wall contains three layers: the innermost endocardium, the middle layer myocardium, and the outermost epicardium. Within these layers, the cellular and ECM composition varies to best suit the functionality of specific cardiac regions. The endocardium possesses a heart chamber thin layer of endothelium and a luminal side of smooth muscle cells and connective tissue fibers. The epicardium is considered the visceral part of the pericardium and consists of a thin transparent layer of mesothelium and connective tissue; it provides a smooth and slippery texture to the heart wall. The myocardium is responsible for the heart’s contraction and propagation of action potential; it is mainly composed of CMs, with a smaller population of non-myocyte resident cells, and a bulk of around 70% of fibrillar collagen I and V (Zhou and Pu, 2016; Gray et al., 2018).

Although a cardiac scaffold with the purpose of repairing and regenerating cardiac damage should include the entire heart layers in cells and matrix compositions, we will focus in this review on tissue equivalents or patches generated by means of cardiac tissue engineering for myocardial reconstruction. Hence, we will analyze below in more detail the structure, function, and composition of the myocardium. Myocardial ECM combines fibrous proteins (collagen, elastin), adhesive glycoproteins (laminin, fibronectin), and proteoglycans to form a complex three-dimensional (3D) architecture that supports cells during contraction (Johnson et al., 2016). Fibrous proteins allow the heart wall to prevent overstretching in multiple directions and contribute to force transmission during contraction. In particular, collagen IV and V, with small amounts of proteoglycans (agrin and perlecan) and glycoproteins (laminin and nidogen), form a basement membrane that surrounds each CM. All this bulk is interconnected with the collagen network and elastin bundles (Bejleri and Davis, 2019). Specifically, collagen I and III and elastin provide respectively rigidity and robustness, compliance, and elasticity to the ECM (Parker and Ingber, 2007). These components contribute to the connection process of the contractile apparatus of adjacent CMs. Lastly, structural ECM includes also mainly proteoglycans–such as biglycan and decorin - and fibrous glycoproteins - such as fibrillin 1 - that are essential for secondary structural support and induction of intracellular signaling. Matricellular components comprise collagen VI, fibronectin, dermatopontin, emilin 1, fibulin 5, lumican, periostin, prolargin, and thrombospondin 2 (Perea-Gil et al., 2018; Bejleri and Davis, 2019).

The fundamental cell type of the myocardium is the CM population, composed of cells with a tubular structure of 80–100 μm length and enriched of chains of myofibrils, i.e., rod-like units within the cell (Duan, 2017). CMs fulfill the tissue contractile function: apart from constituting the principal cellular population of the myocardium, they occupy approximately 33% of cells and 75% of the volume of the whole heart. They are interconnected by a nanofibrous structure that leads to the assembly of the sarcomere, the fundamental contractile unit. Laminar or microfibrous structures reinforced by the collagen network guarantee CM assembly (Torrent-Guasp et al., 2005; Chabiniok et al., 2016). Collagen networks and CMs have a close highly integrated interaction that provides mechanical strength, elasticity, and structural stability to the heart (Torrent-Guasp et al., 2005; Williams, 2008). This architecture also endows the tissue with rapid propagation of signals–including electrical ones-through gap junctions triggered by chemical signals (Chung et al., 2007). Besides working CMs, the myocardium is populated also by specialized conduction CMs, endothelial cells (ECs), vascular smooth muscle cells (SMCs), immune resident cells, and cardiac fibroblasts (CFs) (Zhou and Pu, 2016; Gray et al., 2018). In the normal heart, CFs are responsible for mantling the ECM architecture and heart function. CFs crosstalk with CMs through paracrine, mechano-electrical, and direct electrical signaling in order to maintain homeostasis and remodel the tissue in response to the physiological increase of cardiac load (Brown et al., 2005; Souders et al., 2009). After CF activation, a transient myofibroblast phenotype is acquired that solves two principal functions: secretion of collagen (mainly types I and III) and remodeling of the neo-ECM by contractile forces. Although myofibroblasts participate in homeostasis and cardiac repair, they are the principal cause of fibrosis and negative remodeling (Creemers and Pinto, 2011; Garoffolo et al., 2022; Ragazzini et al., 2022). Myofibroblast activation is an opportunistic and multi-step process that is turned on in many cells to respond to an injury signal (Yu et al., 2018). ECM remodeling is an active process with pleiotropic effects. While it is needed to prevent wall rupture in response to an acute injury associated with extensive CM loss, persistent fibrosis also drives pathology. CF fate is regulated by extracellular cues that activate complex signaling networks and epigenetic modulation, ultimately resulting in modified transcriptional responses (Brown et al., 2005). Accumulating evidence suggests Hippo-YAP signaling modulates CF activation and differentiation and can affect the extent of fibrosis following tissue injury (Del Re, 2022). Increased mechanical stresses are the hallmark of fibrotic remodeling and can result from altered cell-cell contacts, changes in cellular tension, or differences in ECM stiffness (Yu et al., 2018). Enhanced substrate rigidity causes increased assembly of focal adhesion complexes and formation of stress fibers, which elicit Yap/Taz nuclear localization and activity. Thus, mechanical stress, with Yap/Taz activation, induces CF activation and transcription of myofibroblast and contractile genes (pro-collagen, alpha smooth muscle actin, and fibronectin) (Xiao et al., 2018; Yu et al., 2018; Liu et al., 2021a; Del Re, 2022; Ragazzini et al., 2022). Hippo-Yap signaling in adult CMs also plays a critical role in modulating responses to cardiac stress. Physiologically, CMs are exposed to the passive stiffness of the cardiac muscle and the active force of the heart contractile apparatus (Yu et al., 2018). In cardiac disease settings, the active force of the heart changes causing the loss of CMs together with alterations in the cardiac load and output (Brown et al., 2005; Yu et al., 2018). The mechanical stimuli to CMs result in modifications in gene and protein expression and cellular shape. These changes are generally associated with an increase in cardiac stiffness, with the heart tissue becoming less compliant, as observed in dilated or hypertrophic forms of cardiomyopathy (Brown et al., 2005; Yu et al., 2018; Frangogiannis, 2019). Both alterations are associated with impaired contractility and heart failure due to the altered forces produced by CMs. Moreover, the remodeling phase of MI, added to the increase in collagen I and fibronectin deposition, is also associated with a differential expression of integrins (Berry et al., 2006; Frangogiannis, 2019). Therefore, MI fundamentally alters the ECM properties and leads to heterogeneity of collagen deposition and alignment (Brown et al., 2005; Chung et al., 2007; Garoffolo et al., 2022). Disturbed collagen fiber organization not only impairs heart pump function but also local signals to endogenous cells by influencing their behavior (Katz, , 2002; Berry et al., 2006; Yu et al., 2018). MI regions with aligned collagen were found more prevalent at the border zone and exhibited myofibroblast enrichment, more than disorganized collagen typical of the core (Bugg et al., 2020). Therefore, physical factors, ECM remodeling environment, and mechanosensing stimuli should be taken into consideration in the forecast of creating scaffolds or patches for cardiac repair/regeneration.

### 2.3 Tissue engineering of the myocardium: From matrix and cells composition to the ideal scaffold

After having pointed out the relevance of tissue-specific biophysical cues in healthy and pathological hearts, it appears explicit for any tissue engineering effort to rely on a scaffold able to reproduce the matrikine signaling of the physiological native tissue. A fortiori, the heart is composed of several tissues, each of them endowed with specific ECM fibers and proteins with a fundamental supportive scope during each exclusive regional function.

Biomimetic scaffolds have the purpose to provide a suitable cellular microenvironment like the native ECM and emulate its ability to sequester and store growth factors and bioactive proteins. To replicate the appropriate structure and physiological function of tissues, the biomaterials used in these approaches should exhibit properties that mimic those of natural biofunctional interfaces (Bertocchi et al., 2017; Sarangi et al., 2017), as tissue-specific scaffold bioabsorption, functionality, biocompatibility, cellular adhesion, and behavior. Moreover, by mimicking important features of ECM architecture, fine control over nano- or microscale cell stimuli can be operated on ((Nicolas et al., 2020)). Therefore, the rational design of a scaffold should be based on considering ECM as a native scaffold that provides cells with a variety of physical, chemical, and biological cues affecting their growth and function (Dzobo et al., 2018). In this way, it shall be possible to create a favorable microenvironment for neo-tissue formation, with a view to improving the complex issue of repairing and regenerating tissue and, at the same time, maintaining its functionality. Nevertheless, a crucial part of biomimetic scaffold design should be covered by the identification and understanding of native ECM composition and role, considering the specific and characteristic interactions with cells and their integrins receptors (Schwartz, 2010). Indeed, matrix composition and organization are also functional to the mechanical behavior of a given tissue. Therefore, a scaffold should reflect the native tissue ECM, in order to ensure an easier function recovery but also have a sufficed porosity and proneness to appropriate cell repopulation, growth, and differentiation (Abbasi et al., 2020).

Engineered materials allow creating biomimetic stimuli to induce specific cellular responses at these interfaces. When a biomaterial is implanted, its compatibility is also decided by the interaction with the recipient’s tissue, including protein adsorption, cell adhesion, activation of macrophages, and inflammation. Substantive progress in material nanotechnology and chemical synthesis provides improved control over the microenvironmental reconstruction of tissues *in vitro*.

Cardiac tissue engineering aims to remuscularize post-infarct myocardium, support endogenous repair mechanisms, and replace failed tissue. Biomimetically designed materials have been used as structural support for cardiac patches or bioartificial hearts (Shapira et al., 2016). As we will describe later, the engineering approach and the scaffold choice can be manifold depending on the specific therapeutic goal. However, there are general features and requirements strictly necessary in the scaffold design and fabrication independently from any cell types or approaches. The cardiac muscle tissue is considered a composite viscoelastic material made of various cell types surrounded by an ECM network (Tiburcy et al., 2017). It is normally aligned in an anisotropic formation, with the tissue elements organized in a nonlinear spiral that increases the overall strength and creates the proper direction of contraction (Rushmer et al., 1953). Indeed, the cardiac patch design needs to address two primary functions characteristic of the myocardial tissue: electrical conductivity and mechanical contractility.

Usually, traditional tissue engineering approaches focus on the heart’s contractility and structural support, inasmuch conduction of signal is vital to proper function but can be acquired during tissue maturation (Didié et al., 2013). A tissue-like scaffold provides solidity and integrity to the cardiac engineered patch, but also supports the right microenvironment for cells and helps to maintain their behavior over time (Vunjak-Novakovic et al., 2010). In addition, the cardiac ECM helps to sustain other physical factors important for the development of a fully mature cardiac tissue as rigidity and topography, mechanical loading and stretch, shear stress, and electrical stimulation (Gaetani et al., 2020).

Although not a universal method has been created to engineer a cardiac patch, several approaches have been applied in its fabrication, including the generation of hydrogels, electrospun fibers, decellularized native tissue- or engineered cell sheets-derived ECM, and decellularized whole heart tissue scaffolds.

### 2.4 Which is the most suitable scaffold for a biomimetic cardiac approach?

Plain scaffolds are not beneficial alone when the whole myocardial tissue function has to be restored since excitation and contraction can only be provided by the presence of specialized cells. However, they might be used to prevent further adverse remodeling of the ventricular chamber in the case of reinforcement therapy. This approach is one of the first applied in cardiac tissue engineering, and the clinical use of these restraint biomaterials is still valid to prevent any further dilatation, one of the typical, irreversible sequelae of MI (Ghanta et al., 2022).

**FIGURE 2:**
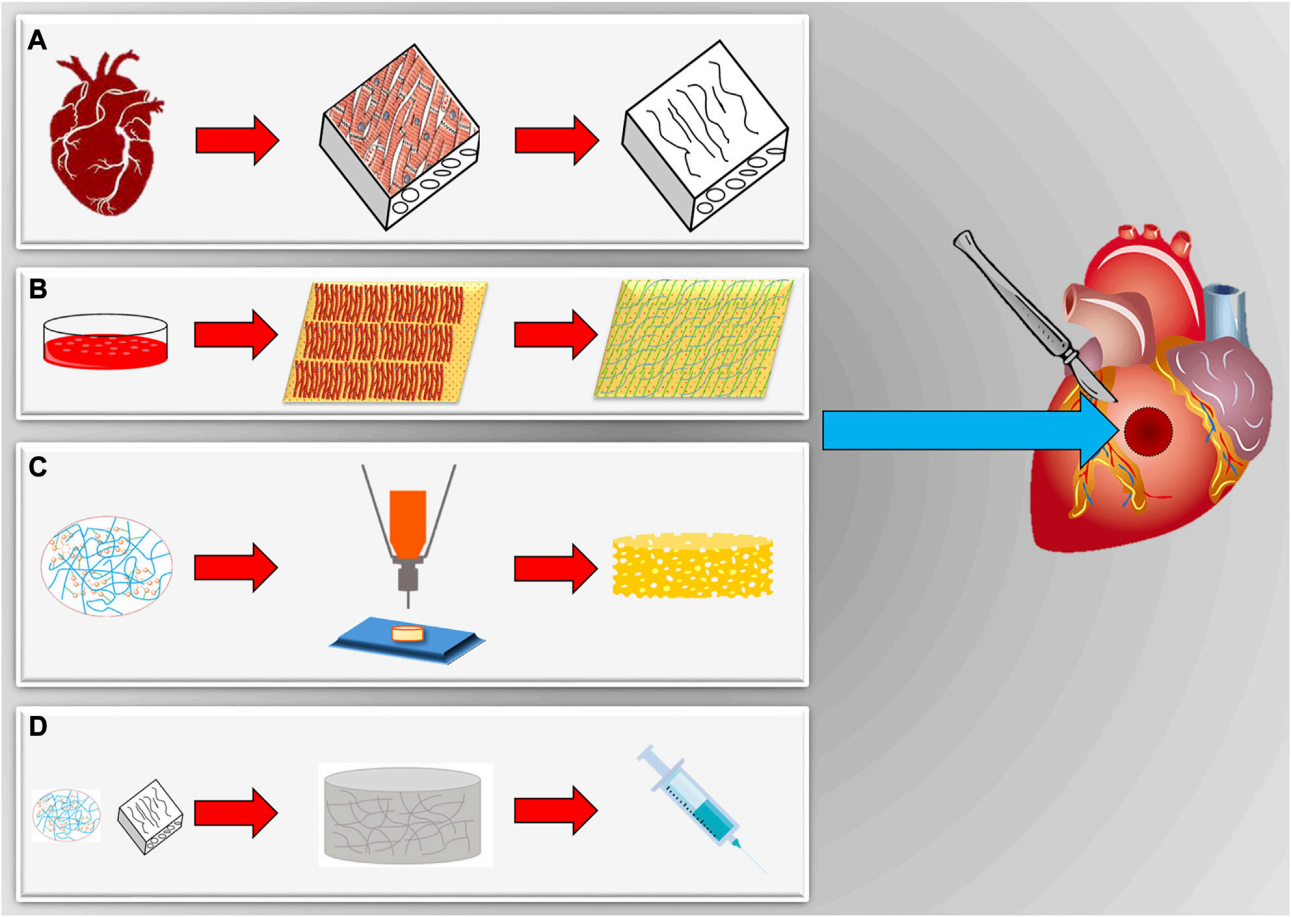
Scaffolds for *in vitro* engineering and *in situ* regeneration of myocardial tissues. **(A)** Cardiac organs can be a source of natural scaffolds after decellularization and isolation. **(B)** Cell sheets submitted to decellularization provide a cardiac-like extracellular matrix synthetized by cardiomyocytes *in vitro*. **(C)** Scaffolds can be also realized with many different biomaterials, both natural and synthetic, and techniques (bioprinting, electrospinning, molding, *etc.*) to tune biomechanical characteristics (elasticity, porosity, *etc.*). **(D)** Scaffolds can be also available as hydrogels, obtained gelatinizing a specific biomaterial. They can assume a solid transition liquid-like state to be injected, also with cells, and afterwards be polymerized in the site of final application.

For the generation of a tissue-engineered cardiac patch as a working myocardial equivalent (Figure 2), the chosen scaffold has not only to provide a hosting environment to single cells but also to support them in the development of a functional syncytium responsible for the excitation-contraction mechanism at the basis of the pump function. Moreover, for MI effective therapy, it is fundamental to restore and guarantee the supply of oxygen and nutrients and reverse/inhibit the adverse remodeling. Synthetic and natural biomaterials have been both adopted in cardiac tissue engineering. Coating, functionalization, or chemical modifications became common technologies to enrich scaffolds for the purpose of specific heart function restoration. The first scaffolds that attracted the interest of the researchers in the field were sponge-like materials with a variable degree of porosity. Microfibrillar collagen I sponges were demonstrated to be a valid starting material in tissue engineering applications after *in vitro* seeding with several cell types, including SMCs, cardiac stem cells, and CMs (Kutschka et al., 2006; Callegari et al., 2007; Chimenti et al., 2011). Being collagen I the most abundant protein in the ECM, this material was already appreciated clinically for other applications, such as adjunctive hemostasis. These porous scaffolds revealed supportive and pro-angiogenic properties that could be enhanced by enrichment or functionalization with vascular growth factors, as VEGF, or peptides, as RGD (Schussler et al., 2021), or combined with synthetic materials, as poly (glycolic) acid (PGA) (Hosseinkhani et al., 2010; Gaudiello et al., 2017). Collagen I is often associated with fibrosis in the myocardium and although very porous in the microfibrillar state of used scaffolds, its relative rigidity might affect not only the spatial distribution of seeded cells but also their ability to connect electrically and mechanically. This is particularly problematic for CMs. Hydrogel formulations were adopted to improve cell-to-cell connectivity (Latifi et al., 2018; Joshi et al., 2019; Schlick et al., 2019; Lu et al., 2021). Their engineering with magnetic nanoparticles or carbon nanotubes further contributed to improving cell alignment and electrical crosstalk (Sun et al., 2017; Yu et al., 2017; Zwi-Dantsis et al., 2020). Besides collagen I, other natural materials found large use to manufacture hydrogels for cardiac applications, as the collagen-originated gelatin, the brown seaweed-derived alginate, the coagulation-related fibrin, the non-sulfated linear glycosaminoglycan hyaluronic acid, the crustacean chitosan, or their combinations (So et al., 2009; Nezhad-Mokhtari et al., 2020; Sisso et al., 2020; Zhang et al., 2022a; Chang et al., 2022; Tohidi et al., 2022; Wu et al., 2023). Hydrogel composites can better replicate the thin and delicate ECM network typical of the myocardium. Hydrogel formulations are deemed particularly versatile in a prospective clinical translation due also to the possibility of injection and controllable polymerization by temperature variation or other physical methods. An interesting comparison by Serafin et al. evidenced the particular physicochemical properties of hydrogels based on the natural polysaccharides gelatin, alginate, and hyaluronic acid in terms of cytocompatibility, porosity, biomechanical performance, swelling degree, chemical structure, and rheological behavior. Among tested combinations, the scaffolds achieved with mixtures of hyaluronic acid and gelatin, or alginate showed both controlled pore size and swelling, especially when chemically crosslinked. The lowest cytocompatibility observed in the presence of alginate can be a double-edged weapon since it could represent a valid coating strategy to overcome inflammatory response after implantation (Serafin et al., 2023). To enhance cell adhesion in coating approaches, gelatin has been combined with methacrylate in the so-called GelMA (Davis et al., 2022) and reinforced in either continuous or discontinuous patterns (Wu et al., 2017; Roshanbinfar et al., 2018; Zwi-Dantsis et al., 2020). Among other glycoconjugates with high translational power, the protein fibrin is particularly attractive for an autologous approach since it can be generated by elements, as fibrinogen, isolated from the patient’s blood (Schaaf et al., 2011; Chang et al., 2022). Apart from polysaccharides and other glycosidic molecules, a biopolymer extracted from the silkworm, silk fibroin, was recently effective for the reconstruction of the myocardial lamellar architecture at the basis of its anisotropy and conduciveness (Mehrotra et al., 2022; Yin et al., 2023). Cardiac cell alignment has also been controlled by electrospinning, modifying the nanotopography and/or micropatterning, and using elastomeric platforms or 3D/4D bioprinting (Cui et al., 2020; Kim et al., 2021; Iwanaga et al., 2022; Ahn et al., 2023; Zhang et al., 2023).

With the purpose to improve the electrophysiological properties of cardiac engineered constructs particularly based on electrically insulating natural hydrogels, conductive materials were developed to facilitate impulse propagation. Polymers (polyaniline and polypyrrole) and materials (MXene, graphene oxide, carbon nanotubes, gold nanorods/wires, poly(3,4-ethylenedioxythiophene):polystyrene sulfonate, silicon carbide nanowires, polyvinylidene fluoride, *etc.*) were selected for their semiconductor or piezoelectric behavior to improve electrophysiological differentiation and synchronize the beating of cultured CMs and their precursors (Dvir et al., 2011; Zhang et al., 2017a; Tomecka et al., 2017; Ye et al., 2020; Alegret et al., 2022; Lagonegro et al., 2022; Mancino et al., 2022; Zhao et al., 2022; Ul Haq et al., 2023; Wu et al., 2023; Yin and Jiang, 2023). Of interest, various of these materials have been combined with bioinspired adhesive proteins derived from mussels, as dopamine used a crosslinker to reach improved superelastic and mechanical features of scaffolds based on polypyrrole or MXene (Wang et al., 2016; Ye et al., 2020).

With inspiration from biological processes, several smart materials were generated by biofunctionalization or encapsulation to maximize the adhesion of specific cells and spatiotemporal control of the homing process (Lai et al., 2014; Chang et al., 2022; He et al., 2022; Yang et al., 2022), facilitate delivery and imaging (Sun et al., 2022), improve oxygenation (Suvarnapathaki et al., 2023), as well as reduce oxidative stress and inflammation, regulate autophagy and/or ameliorate cardiac function (Shilo et al., 2021; Fu et al., 2022; Guo et al., 2022; Wu et al., 2022).

With similar final aims but diverse strategies, a particular class of biomaterials with thermoresponsive properties have also been utilized to construct sheets of different cells. By lying on these films, cells are allowed to grow while creating their intracellular junctions, possibly synthesizing novel ECM fibers to establish anchoring and communication. After the so-called sheet has been created, a trivial shift in temperature is sufficient to modify the hydrophobic state of the biomaterial and detach it. As such, the biomaterial is only ancillary to generate the cell sheet and is removed before any *in vivo* application. Thermoresponsive polymers, as poly(N-isopropylacrylamide), found use to generate sheets of CMs, CFs, SMCs, ECs, and so on. Differently from other biomaterials-based strategies, this approach has the unique advantage of generating a complete tissue by combining several cell sheets, including the ones needed to reconstruct a vascular net able to prevent inadequate perfusion and core necrosis (Masuda et al., 2015; Iwamiya et al., 2016; Kawamura et al., 2017; Nagase et al., 2017; Nakayama et al., 2021a; Nakayama et al., 2021b). Intriguingly, thermoresponsive polymer technology has been demonstrated to be a useful tool to create biological pumps by shaping multilayered cell sheets in beating tubes or ventricles (Mohammadi et al., 2022; Sekine and Okano, 2022).

Reproducing a physiological ECM with synthetic polymers or combinations of natural materials represents a never-ending challenge, at least with the current state-of-the-art of knowledge and technologies. As aforementioned, the physiologic scaffolding of a tissue is a complex, 3D, and tissue-specific network of fibers, proteins, and glycoconjugates. It is probably characterized by components, whose presence, combination, and/or distribution might still be incompletely known and very dynamic, and thus it is demanding to fully recapitulate artificially. A modality to overcome these limitations is the usage of native tissue and organs, whose ECM is isolated by the removal of the resident cells through a process termed decellularization. In opportune conditions, decellularized scaffolds preserve the original ECM of tissues and organs, including the network supporting their vasculature and innervation. Such a scaffold type is ideally derived from human donors. Indeed, to overcome donation paucity, an acceptable alternative considers animals sharing similar anatomical and physiological characteristics, for example, pigs. Decellularized scaffolds are used in their solid state or can be transformed in hydrogels for patch applications, as well as being intravascular infused, generally revealing optimal homing capabilities for seeded or recruited cells (Ott et al., 2008; Wainwright et al., 2010; Sarig et al., 2012; Dal Sasso et al., 2020; Spang et al., 2022).

## 3 The shortening gap between preclinical research and clinical application of cardiac engineered tissues

### 3.1 Different applications of cardiac tissue engineering in regenerative medicine and surgery

Cardiac damage can result from various insults and brings to significant loss of viable myocardium, in turn leading to pathogenetic mechanisms involving neurohormonal dysregulation, hemodynamic overload, cardiac remodeling, abnormal calcium cycling, and ECM dysfunction (Braunwald, 2013; Sorriento and Iaccarino, 2019). After a MI event, the heart undergoes an inflammatory stage characterized by immune cell infiltration and granulation tissue formation. During the resolution of inflammation, myofibroblasts modify healthy, aligned fibers with new collagen deposition, resulting in thin hypocellular fibrotic tissue (Virag and Murry, 2003; Prabhu and Frangogiannis, 2016). The formation of fibrotic\scar tissue combined with the death of viable CMs results in the loss of mechanical contraction capacity, often measured through the left ventricle ejection fraction (LVEF) (Zhang et al., 2013).

As the physiological myocardium possesses a hierarchically aligned structure with different layers, materials with micro- and nanotopography and structures have also been used to direct the alignment of CMs within the engineered tissue through anisotropic bio-matrix interfaces, aiming at restoring physiological electrical conduction (Bursac et al., 2007; Kim et al., 2010; Ren et al., 2017). Following transplantation, the scaffold should maintain local mechanical compliance preventing fibrosis activation due to emerging stiffness-sensitivity of myocardial resident cells. Scaffolds offering a 3D, perfusable cardiac-like ECM reduce the risk of negative mechanosensing activation and immunological post-transplant reactions. These interfaces should provide also microenvironments promoting *in situ* cell migration and proliferation (Lu et al., 2013), as well as appropriate electromechanical coupling and stable contractility (Leor et al., 2000; Zimmermann et al., 2006). This requires a fine material/cell combination and tuning to recapitulate the physiology of the normal tissue environment and promote a stable patch engraftment coordinating or integrating with the action potential propagation of the recipient’s myocardium (Bursac et al., 2007). Generally, biomaterials with high cell homing and guiding abilities are poor electrical conductors and block signal propagation in the cell-matrix interface, by limiting the capability of the patch to contract strongly as a unit. As before mentioned, the incorporation of conductive nanoparticles into CMs-seeded scaffolds enhanced the conductive properties of cell-polymer-cell interfaces (Dvir et al., 2011; You et al., 2011), by bridging the electrically resistant polymer and improving electrical communication between adjacent CMs. Moreover, folded 3D nanoelectronic scaffold/cardiac tissues provided electrically conductive interfaces to record extracellular action potentials in real-time (Dai et al., 2016). Such conductive cardiac scaffolds also improved cell-to-cell coupling due to the increased expression of the Connexin-43 gap junction protein, reduced dispersion in CM repolarization, and facilitated electrical propagation and synchronous contraction (Vunjak-Novakovic et al., 2010; Yasui et al., 2014). Eventually, the cardiac patch should support the presence of a functional vasculature. In fact, the myocardial tissue requires large amounts of oxygen and nutrients to ensure a continuous function throughout the lifetime of an individual. This feature is strictly related to scaffold porosity that might allow ingrowing endogenous vasculature (Abbasi et al., 2020). Regulating micro- and nano-porosity and structural cues may provide patterns that favor angiogenesis and guide blood vessel formation (Wang and Ho, 2004). Nevertheless, porosity is difficult to control, and, when high, it could negatively affect the scaffold mechanical structure. A usual choice is the utilization of scaffolds with grooves’ dimensions corresponding to desired vessel size. In addition, mechanical cues, such as the shear stress of the blood against the vessel walls, could enhance vascularization. Shear stress is commonly considered the *primum movens* in vasculature development. When exposed to flow, cells and stress fibers align accordingly and several key angiogenesis pathways are activated (Tandon et al., 2009). Due to the variety observed in physiological and pathological vascular settings, shear stress is difficult to be accurately reproduced *in vitro* (Wang et al., 2017).

Tissue engineering continues to expand as a scientific framework to advance cardiac regenerative medicine. Three categories can define cardiac tissue engineering products: cell-free scaffold approaches, scaffold-free cellular approaches, and cell plus scaffold hybrid approaches (Valdoz et al., 2021).

#### 3.1.1 Cell-free scaffold approaches

The native ECM is not an inert scaffold that only provides mechanical support for the cells. Rather, it is involved in essential signaling pathways that regulate key cellular functions [(Holmes et al., 2005; Kolewe et al., 2013)], such as adhesion, biophysical cues, cell alignment and polarity, and tissue stiffness. As discussed above, the integrin family regulates cell-ECM interactions and downstream signaling pathways. Considering the final purpose of tissue replacement, mimicking cardiac ECM is one of the tissue engineering approaches that have the promise to rejuvenate the myocardium and improve contractility. Scaffolds-based therapeutic approaches target inelastic, stiff myocardium, with the optimum of ECM tissue reconstruction in terms of durability, conduction, and elasticity. To provide these essential cell-ECM interactions and develop functional cardiac tissues in engineered myocardial patches, different strategies have been adopted. Indeed, tissue-engineering strategies have pursued both natural, synthetic, and decellularized materials to recreate healthy ECM in preclinical studies intended to repair the dysfunctional myocardium (Figure 2).

##### 3.1.1.1 Cell-free scaffolds

The importance of acellular grafts has been evaluated as mechanical–structural support for MI heart. Acellular scaffolds possess several advantages over recellularized ones because of their off-the-shelf availability for immediate implantation, extended shelf lives, better mechanical and functional properties, and limited immune reactions (Lu et al., 2004). Biomaterials, including hydrogels and elastomers, allow creating scaffolds of various porosity and stiffness (Meier and Hay, 1975; Frey et al., 2014; Tomov et al., 2019) to better develop and integrate into the myocardium with optimal electrical conduction and biodegradability (Martens et al., 2009). In a clinical trial, a bioabsorbable cardiac matrix was tested for its suitability in attenuating left ventricular remodeling after a large MI. This approach utilized a device replacing the damaged ECM and showed the ability to halt the remodeling process following acute ischemic damage (Shin et al., 2013; Rao et al., 2016). In the work of Serpooshan et al., an engineered acellular collagen patch was transplanted into the infarcted myocardium of adult murine hearts. The physiological outcomes were evaluated 4 weeks post-MI and compared with the control group. According to the collected outcomes, acellular cardiac engineered tissues were repopulated with host native cells (CFs, SMCs, epicardial cells, and immature CMs) and integrated with the recipient’s tissue. They contributed to preserving contractility, reducing left ventricular remodeling, and suppressing the onset of fibrosis (Serpooshan et al., 2013). Shah et al. examined the therapeutic effect of acellular cardiac patches derived from decellularized porcine myocardium. After implantation, they observed firm attachment of cardiac slices to the host myocardium and robust cellular infiltration. Notably, a high density of M2 pro-regenerative macrophages, as well as significant neoangiogenesis were detected (Shah et al., 2019).

##### 3.1.1.2 Decellularized cell sheets

Although a decellularized native tissue provides a highly biomimetic scaffold with structural and compositional details closer to native ECM, several roadblocks remain in this approach, including scarcity of donors, huge batch-to-batch variations, possible pathogen transfer, and undesirable immune responses toward allogenic or xenogeneic ECM or cell remnants. The application of species- and tissue-specific, decellularized cell layers, grown from pathogen-free cells, can potentially avoid these issues while serving as a more biomimetic microenvironment. CFs are one of the suitable cell sources for this aim. Schmuck et al. characterized the ECM composition of a decellularized rat CF sheet and observed a distinctive content: 82% fibronectin, 13% collagen type I, 3.4% collagen type III, 0.2% collagen type V, and 1.3% elastin, along with 18 non–structural bioactive molecules (Schmuck et al., 2014). Due to the high fibronectin content, these scaffolds are easy to attach to native epicardium without glue or sutures. CFs can be obtained by means of a heart biopsy and are easier to expand than isolated CMs, as demonstrated by Pagano et al., who obtained decellularized ECM from CFs harvested from the right atrial appendage of healthy or diseased human hearts (Pagano et al., 2017). For structural and functional maturation of cardiac cells in an engineered patch, it is also crucial to develop in-built vasculature scaffolding that can support graft recellularization and survival after implantation. Several pre-vascularization strategies have been developed to date to form functional channels within 3D tissues, which can be anastomosed with the host after implantation (Dvir et al., 2009; Noguchi et al., 2016).

##### 3.1.1.3 Injectable scaffolds

Injectable hydrogel technology allows for assembling a 3D polymeric network with a high-water content. This technology is extensively used in tissue engineering approaches or systems for the delivery of therapeutic agents and cells thanks to the high permeability, biodegradability, and biocompatibility of these hydrogels. These biomaterials can be easily modified to provide specific physical, chemical, and electrical properties. In particular, they can support the conductive properties of the heart (Camci-Unal et al., 2014; Frey et al., 2014; Hasan et al., 2015) and varying stiffness compatibility with constant contractions (Khan et al., 2016), making them highly compatible with the cardiac environment. Although patch-based and cell sheet systems have been widely studied and offer promising results for cardiac tissue engineering, they require more invasive surgical intervention. These procedures may be more complicated to translate into clinical applications, in which minimally invasive techniques are preferable (Li and Guan, 2011). Because injectable hydrogels can be deployed into the myocardium through minimally invasive approaches, such as catheter delivery, they are particularly appealing for cardiac regeneration (Moorthi et al., 2017). In a clinical pilot study, Frey et al. tested an injectable acellular alginate-based hydrogel on patients affected by MI. No patients were reported to undergo adverse events and LVEF was preserved. These results make injectable hydrogels promising materials for such cardiac regeneration therapeutical purposes (Sepantafar et al., 2016). Finally, hydrogels could be injected directly into the damaged cardiac tissue as a potential vehicle to deliver cells, growth factors, and therapeutic peptides or drugs (Shu et al., 2015; Ungerleider et al., 2015). They may also be used to support various gene delivery systems enabling controlled expression for a more efficient therapy (Nishita et al., 2017; Shafei et al., 2017).

#### 3.1.2 Cell-based approaches

**FIGURE 3:**
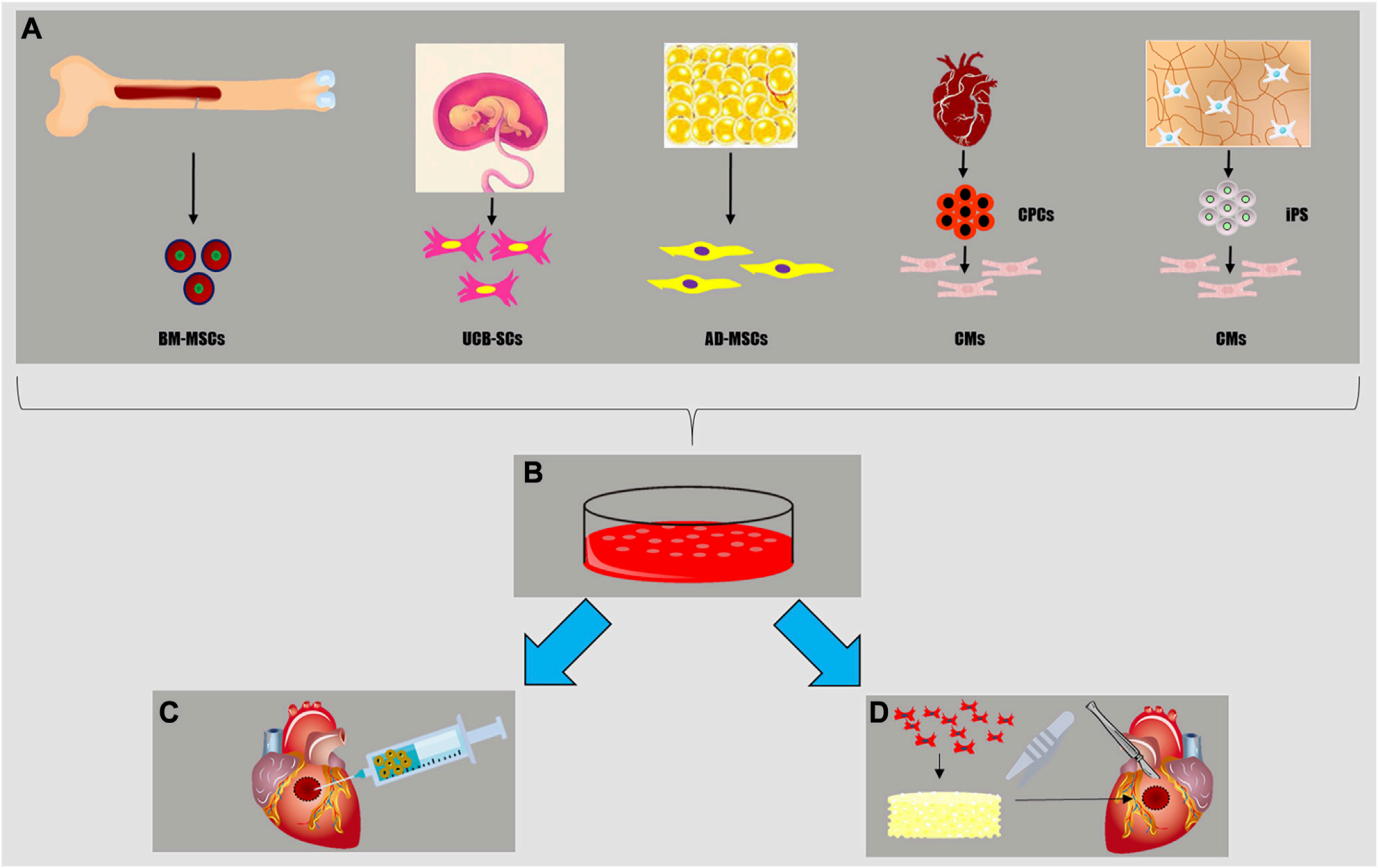
Cell sources for myocardial tissue engineering **(A)**: mesenchymal stem cells from bone marrow (BM-MSCs), stem cells from umbilical cord (UC-SCs), adipocytes and adipose-derived mesenchymal cells from adipose tissue (AD-MSCs), cardiomyocytes (CMs) either differentiated by cardiac progenitor cells (CPCs) or induced pluripotent stem cells (iPS). Once isolated, these cells can be cultured *in vitro* **(B)** in order to realize a cell-sheet, a sufficient number for an *in situ* heart injection **(C)**, to populate a scaffold creating a viable myocardial tissue patch **(D)** for *in vivo* implantation or *in vitro* disease modeling and drug testing.

Recent treatments for MI and chronic heart failure are centered on recellularizing the myocardium and inducing repair and regeneration of the injured tissue (Wang et al., 2010). The most basic approaches rely on bolus injections of dissociated stem/progenitors and terminally differentiated cells via various delivery routes, such as intracoronary and intramyocardial infusions (Golpanian et al., 2016). Cell-based cardiac treatments attempt to stimulate the heart’s self-regeneration ability by adding paracrine signaling cues and repopulating the damaged tissue with new healthy cells, hence improving the overall function and structural integrity of the myocardium (Guo et al., 2020). Target cells are chosen from various sources with reference to two main criteria: first, their ability to recellularize the damaged myocardium based on their proliferative and differentiation capacity; and second, their availability and abundance for easy harvesting and expansion *in vitro* (Figure 3).

Cell sheet technology is one of the cell-rich strategies widely used in cardiac tissue engineering and regenerative medicine. Cell sheet tissue synthesis, as a scaffold-free self-assembly method, is utilized to create 3D living constructions (Masuda and Shimizu, 2016). The cell sheet might have several cardiovascular uses, including as a unique cardiac patch for repairing myocardial defects. In particular, two studies showed these types of cardiac patches guarantee excellent vascular integration, CM proliferation, and improved cardiac function (Matsuo et al., 2015; Masumoto and Yamashita, 2016). Cell sheets-based bioengineered tissues do not induce inflammatory responses or fibrosis, because they do not contain biodegradable materials, are non-thrombogenic, and grow in proportion to the host cardiac tissue (Kobayashi et al., 2008; Masuda and Shimizu, 2016). Cardiac cell sheets have been expanded by various cell types (Shimizu et al., 2006; Matsuura et al., 2009; Miyagawa et al., 2010; Baikova et al., 2011; Masumoto et al., 2012; Masumoto et al., 2014; Zhang et al., 2015; Kawamura et al., 2017; Dergilev et al., 2018; Roberts et al., 2019; Theus et al., 2019) and their transplantation is nowadays the most extensively researched application, with several promising results in models *in vitro* and in experimental animals *in vivo* [(Bertocchi et al., 2017; Nicolas et al., 2020)]. This regenerative strategy is not only preventative but also curative in terms of new CM reserve and blood vessel formation. Furthermore, myocardial cell sheet transplantation has previously been evaluated in multiple clinical trials on patients affected by MI and coronary artery disease [(Dzobo et al., 2018; Schwartz, 2010)]. The most promising preclinical and clinical applications of cell sheet technology have been realized by taking advantage of several cell types, as for instance cardiac progenitor cells (CPCs), mesenchymal stem cells (MSCs), bone marrow (BM-) or adipose-derived (AD-), umbilical cord stem cells (UC-SCs), and human pluripotent stem cells (hPS) (Supplementary Table 1).

The adult heart contains resident CPCs, which are a multipotent population of cells that can differentiate into CMs. These cells release paracrine factors with angiogenic and cardioprotective properties, allowing them to migrate and engraft into the damaged area and promote heart regeneration and function. Preclinical and clinical studies have shown that CPC-CM cell sheets can treat cardiac heart failure efficiently. Electrical and functional connections have been demonstrated to develop between the transplanted CM sheet and the recipient’s heart, by restoring the function of the injured organ. Kamata et al. showed that human CPC-CM sheets integrated with the existing myocardium in a swine chronic ischemic model and increased the wall thickness of the LV, ultimately improving LVEF and global cardiac performance (Kamata et al., 2014).

MSCs have been isolated from various tissues and organs. Considering the capability of ameliorating interstitial inflammation and fibrosis, as well as improving cardiomyogenesis and vascularization, MSCs were used for the prevention or treatment of cardiac diseases. MSCs release growth factors and cytokines driving angiogenesis, ECM remodeling, and proliferation, migration, and differentiation of endogenous cardiac stem cells in the injured cardiac tissue. These events are critical in restoring heart regeneration and function following acute and/or chronic diseases. MSCs isolated from the bone marrow are the most extensively utilized stem cells in heart regeneration. Numerous studies have demonstrated the favorable impact of these MSCs in regenerating the myocardium *in vitro* and *in vivo* and ischemic hearts better preserved left ventricular contractility after damage (Matsuo et al., 2015; Bao et al., 2017; Luger et al., 2017; Zhang et al., 2021). A significantly lower infarct size was documented after treatment with these cells rather than with their adipose tissue-derived counterpart (Braunwald, 2018). MSCs derived from the bone marrow did show a trivial ability to develop into functional CMs within injured tissue; rather, their paracrine action aids myocardial regeneration (Li et al., 2009; Karpov et al., 2013; Choudry et al., 2016). The predominant therapeutic effect of these cells in the injured heart consists of the secretion of angiogenic and antiapoptotic factors stimulating the resident cells. MSCs cellular sheets showed potential in the improvement of left ventricular function 1 month after transplantation in an aged rabbit MI model. Their application enhanced angiogenesis in the peri-infarcted area, inhibited apoptosis, decreased the infarcted area in the damaged cardiac tissue (Narita et al., 2013), increased initial retention, and amplified subsequent paracrine effects to recover damaged heart, compared with intramyocardial injection of a single cell suspension (Tanaka et al., 2016). Pre-adipocytes and AD-MSCs showed differentiation potential into contractile CMs (Jumabay et al., 2009; Kim et al., 2021). In the infarcted heart, AD-MSCs sheets increased cellular integration and vascularization while reducing interstitial inflammation and fibrosis. As a result, they improved heart remodeling and function. Some studied examples were based on cell sheets fabricated with AD-MSCs overexpressing SCF, which induced c-kit-positive cells into functional cardiac muscle when transplanted onto ischemic tissues (Miyahara et al., 2006; Yamamoto et al., 2018). The umbilical cord is a rich source of stem and progenitor cells, such as UC-SCs. They have several advantages over bone marrow MSCs, such as higher proliferation, easy harvesting, decreased risk of unsuccessful grafting, enhancement of angiogenesis, and anti-fibrotic effects. Moreover, they possess low immunogenicity (Fonoudi et al., 2015; Afjeh-Dana et al., 2022). Guo and others transplanted UC-SCs sheets post-MI in a mouse model. These sheets dramatically improved cardiac function by promoting angiogenesis, modulating the inflammatory response, and reducing fibrosis (Guo et al., 2021a). In a clinical trial, UC-SCs derived from the cord stroma were also evaluated. A favorable effect was described regarding scar tissue reduction and ventricular wall function restoration, indicating potential efficacy in the therapy of chronic ischemic cardiomyopathy (Ulus et al., 2020).

The cardiac differentiation of hPS, including human embryonic stem cells (hES) or induced PS (iPS), introduced a novel cell source for cardiovascular tissue engineering approaches. The sequential steps of guided differentiation employing a combination of growth factors and small chemicals propel hPS toward CMs (Jung et al., 2012a; Biagi et al., 2021). The therapeutic effect of iPS-CMs has been demonstrated in several small and large animal models of MI, such as mice, rats, and pigs (Ye et al., 2014; Mattapally et al., 2018; Ishida et al., 2019; Jiang et al., 2020; Biagi et al., 2021; Kawaguchi et al., 2021). Researchers used hPS-CMs-cell sheets for cardiac repair in MI models (Masumoto et al., 2014; Kawamura et al., 2017). The transplantation of iPS-CMs sheets reduced fibrosis while increasing *de novo* vascularization and cardiomyogenesis via paracrine mechanisms. Moreover, when self-beating cell sheets made of CMs, mural cells, and ECs differentiated from hPS were transplanted into MI animals, functional and electrical recovery was achieved due to *de novo* vascularization and extended CM retention (Masumoto et al., 2014; Kawamura et al., 2017). In a rat model, Miki and others implanted bioengineered myocardial sheets, generated from mouse iPS, and observed improved cardiac function and chronic MI remodeling attenuation (Miki et al., 2012). Chong et al. reported noteworthy outcomes on the use of ES-CMs sheets in a non-human primate model of myocardial ischemia-reperfusion (pigtail macaque) that resulted in remuscularization of heart tissue but insufficient cell maturation (Chong et al., 2014). Finally, the first world transplant of hiPS-CMs sheets was performed on 10 patients suffering from ischemic cardiomyopathy. The team of Professor Sawa from Osaka University in Japan, who performed this pioneering clinical work hypothesizes that cell sheets might adhere to the surface of the patient’s heart and, through cell secretion, repair blood vessels, and restore cardiac function (Cyranoski, 2018; Miyagawa et al., 2022).

#### 3.1.3 Cellularized implantable scaffolds

The first tissue engineering approaches for MI treatment had the goal of developing *in vitro* functional cellular constructs that could be easily incorporated into the host myocardium *in vivo* (Hirt et al., 2014). The advancements in microscale technologies (i.e., microengineering) in the past few years have provided a unique ability to develop biomimetic tissue models with native-like properties and cellular/ECM organization for regenerative medicine and disease modeling applications (for example, cancer) (Huh et al., 2012; Veldhuizen et al., 2019). Indeed, as pointed out before, the structural anisotropy and architecture of the myocardium are as critical as the cellular and ECM composition of the tissue, by modulating physiologic homeostasis and function. Therefore, the induction of precise cellular organization and architecture, often in conjunction with modulated electromechanical cues, is a crucial point in injured heart tissue treatment (Nikkhah et al., 2012).

In cellularized scaffold approaches, the matrix might imitate the original tissue ECM, providing mechanical support for cells to undergo morphogenesis and assemble in a 3D architecture. Engineered scaffolds can also have integrated architectural and topographical characteristics (Rajabi-Zeleti et al., 2014; Truong et al., 2019a). In addition, mechanical characteristics (stiffness, swelling, and cross-link density) of the scaffolding matrix also have a strong impact on cell behavior, possibly inducing non-physiological phenotypes (Zong et al., 2005; Truong et al., 2019b). A delicate balance between degradation rate and cellular interconnection and deposition of new ECM needs to be achieved. If the scaffold degrades too quickly, the delivered cells will not have the opportunity to form sufficient cell-to-cell and cell-to-ECM interactions to support their engraftment. On the other hand, if the scaffold does not degrade fast enough, it can elicit a foreign body reaction and fibrotic encapsulation (Zorlutuna et al., 2012). To exert strong control over this variable, a first approach consists in repopulating with autologous cells an allogeneic decellularized native tissue preserving architecture and mechanical properties (Chen et al., 2015; Gao et al., 2017). For the first time, Ott et al. described the decellularization of a whole rat heart that was reseeded with CMs and ECs, by obtaining good contractile properties, but nearly insufficient to serve as functionally normal. This approach has the benefit to exploit the native ECM composition and distribution to reconstruct the natural vascular network and permit the exchange of nutrients, paracrine factors, and oxygen throughout the tissue (Ott et al., 2008).

Another possibility is to recreate *ex novo* a biomimetic scaffold using biomaterials (natural or synthetic), tuning its characteristics, and then populating it *in vitro* with cells before implantation. To be successful, the designed cell-scaffold myocardial equivalent must offer the appropriate homeostatic cues to promote vascularization, preserve muscle tissue, and minimize fibrous tissue formation that could hinder contractility and cardiac output. Gao et al. created a crosslinked, GelMA 3D scaffold to mimic fibronectin. Seeded with different hiPS-derived cells (CMs, SMCs, and ECs), these human cardiac muscle patches were tested on adult mice after surgically induced MI. Cardiac function, vascularization, and scar size were investigated. Following quantitative PCR measurements, 24.5% ± 2.6% of the transplanted cells remained engrafted in the hearts; moreover, histology analysis revealed an engraftment rate of 13.6% ± 2.2% after 7 days of implantation. Cardiac function was evaluated on day 28 after injury via echocardiographic assessments of LVEF and fractional shortening: both parameters were significantly greater for animals implanted with cellularized scaffolds (Gao et al., 2017). Qian et al. used highly aligned decellularized human dermal fibroblast sheets as ECM scaffolds to coordinate the physiological alignment of microvascular networks in the co-culture of human MSCs and ECs with great potential for cardiac tissue engineering. hMSC-EC co-culture promoted the secretion of pro-angiogenic growth factors and matrix remodeling via metalloprotease-2 activation, which resulted in highly dense vascular network formation with intercapillary distance (20 μm) like the native myocardium (Qian et al., 2019). Zhang et al. applied an alternative and promising method for constructing vascularized tissues using engineered 3D microvascular tissues incorporating HUVEC cells with alginate-chitosan microcapsules in an ECM collagen scaffold. It was observed that such microcapsules are able to enhance vascular network formation by providing support for cells and guiding cell alignment/organization (Zhang et al., 2017b). In the research by Chen et al., microgrooved collagen scaffolds engineered with myoblasts were proposed as an organized multilayered muscle tissue for implantation to repair/restore the function of diseased tissues. Highly aligned and multilayered muscle bundle tissues were engineered by controlling the size of microgrooves and cell seeding concentration. In the engineered muscle tissue, myoblasts were well aligned, showing high expression of myosin heavy chain and synthesis of muscle ECM (Chen et al., 2015). Hosoyama et al. developed differently nanoengineered, collagen-based electroconductive cardiac patches for infarcted myocardium repair. Those containing spherical nanogold were able to increase connexin-43 expression in neonatal rat CMs cultured *in vitro* under electrical stimulation. After *in vivo* implantation, only nanogold-containing patches were able to recover cardiac function. Histological analysis confirmed a boost in connexin-43 levels, as well as blood vessel density with a reduction in scar size only for animals that received the nanogold patch (Hosoyama et al., 2018). Maidhof et al. advanced the use of porous elastomeric scaffolds in polyglycerol sebacate. These scaffolds are characterized by an array of channels useful to generate conduits for medium perfusion and sized to provide efficient transport of oxygen to the cells (myoblasts and ECs) by a combination of convective flow and molecular diffusion over short distances among the same channels. Perfusion seeding of single scaffolds with rat aortic ECs resulted in preferential cell attachment at the channel surfaces (Maidhof et al., 2010). Sridharan et al. proposed a tissue-engineered construct composed of aligned coaxial nanofibrous polycaprolactone core and gelatin shell, seeded with hiPS-CMs. These nanofibrous scaffolds could have a potential application in the generation of functional cardiac patches for myocardial repair applications as well as an *in vitro* 3D cardiac tissue model to evaluate the efficacy of cardiovascular drugs and cardiac toxicities (Sridharan et al., 2021).

To date, some Phase I clinical trials were performed and provided valuable results. Menasché et al. focused on the treatment of ischemic cardiomyopathy heart failure using a fibrin patch seeded with hES-derived cardiac progenitors (Menasché et al., 2018). For affected chronic patients, a therapy based on a collagen scaffold enriched with MSCs was tested (He et al., 2020). In another randomized clinical study of coronary revascularization surgery, the injection of MSCs and placement of an epicardial ECM were realized [clinical trial NCT04011059 (Randomized)]. In patients with critical conditions and several comorbidities, functional improvement might be difficult to achieve, and, thus, underestimation of real therapeutic potential can occur. However, with respect to other approaches, the clinical utilization of a cell-scaffold combined therapy is expected to enhance myocardial regeneration, reduce scar tissue, and improve cardiac output. Researchers will get a deeper knowledge of certain scaffold materials or cellular therapies based on the findings of these trials, which will provide optimism for future investigations.

### 3.2 *In Vitro* disease modeling with cardiac engineered tissues

#### 3.2.1 Cardiac disease modeling

##### 3.2.1.1 Typologies of cardiac tissue disease models

Disease modeling is indispensable when more information has to be acquired on a given pathology or when an effective or improved therapeutical treatment has to be identified. Several preclinical models were generated to simulate the myocardium and its electromechanical components in physiology and pathophysiology. Heterologous expression systems, 2D cell culture, *in silico* computational models, and *in vivo* animal models are the main approaches used to study cardiovascular pathology and/or the related pathophysiological mechanisms (Iop, 2020). As previously reviewed, these models have contributed to providing relevant insights on the human diseases affecting the heart; however, they have generally failed to offer their completely *bona fide* replicas. The genetic engineering of eukaryotic cells, such as yeasts or non-cardiac immortalized cells, to overexpress human genes has been a widely utilized tool to study the specific interactions of ion channels and/or proteins related to cellular junctions, either in their normal pattern or in the mutated forms distinctive of the cardiac disease. With such an approach, it is possible to exclude any genetic redundancy or interaction with other molecular players the studied protein communicates in the usual environment. This advantage becomes, however, a limit when the effect of the mutation has to be studied on a systems biology level. This approach has therefore to be integrated into a multimodal strategy, often considering *in vitro* modeling with primary cell lines or *in vivo* studies with animals. Very frequently, animals, especially the most adopted rodents, display anatomical and electrophysiological differences with respect to humans that render them only partially useful to model human heart diseases. *In vivo* studies employing transgenic rodents or combined gene/cell therapies in other mammals often fall short in faithfully replicating human physiology, pathophysiology pathways, and intricate interplays involved in various diseases, primarily due to differences in anatomic, metabolic, and electrical signaling functions (Capel et al., 2015; Golovko et al., 2019; Kalyanasundaram et al., 2019).

Several protocols were developed to isolate CMs from human tissues. In the diagnosis of several cardiac diseases and the surveillance of the immunological response to heart transplantation, an endomyocardial biopsy is a fundamental tool to better investigate the pathophysiological culprit. This invasive procedure generally allows for obtaining small tissue fragments, which can be utilized for histopathological and molecular diagnostic analysis. This can be also the only source to derive primary cell lines. However, obtained CMs are unable to proliferate and are unstable due to their physiological peculiarities and tendency to lose their phenotypic characteristics during *ex vivo* culturing. Moreover, the procedure might put the cardiopathic patient at risk (Cooper, 2008; Cooper, 2013) and the final yield in cell population density might be not sufficient for the purposes of disease modeling.

The avenue of hiPS and their potential to differentiate into functional CMs, encompassing atrial, ventricular, and nodal cells, has paved the way for the development of improved human cellular models suitable for drug screening. These models aim to address the limitations of existing preclinical alternatives and improve translational outcomes (Jung et al., 2012a; Iop et al., 2021; Naumova and Iop, 2021). Currently, many *in vitro* pharmacological platforms rely on 2D cell culturing techniques, where cells are grown on plastic Petri dishes. Despite the relatively immature phenotype of hiPS-derived CMs, several of these models have demonstrated their superiority in recapitulating the diseased phenotype and evaluating personalized pharmacological treatments for cardiac disturbances (Jung et al., 2012a). However, it is increasingly recognized that incorporating cell-ECM constructs enhances cell maturation and provides a more accurate representation of the complex 3D microenvironment and architecture of the tissues. Besides improving physiological relevance, 3D modeling of cardiac diseases offers several other advantages for more accurate emulation of the functionality of the human myocardium (Zimmermann and Eschenhagen, 2007; Tulloch et al., 2011). It facilitates the study of cell-cell interactions, such as gap junctions and cell signaling networks, and simulates tissue-level phenomena, such as electrical conduction, mechanical contraction, and vascular perfusion, allowing to capture processes impossible to replicate in 2D models (Zimmermann and Eschenhagen, 2007; Tulloch et al., 2011). It also allows for a deeper investigation of drug screening and personalized medicine to better predict efficacy and toxicity. Precisely predicting cardiac toxicity in preclinical settings is a formidable challenge. Despite rigorous pharmacological testing, many drugs that show promise in animal models fail in human therapies due to cardiac toxicity or functional alterations. As such, 3D tissue models engineered with human cells are anticipated to fill the gap between preclinical research and clinical translation. Cardiac tissue engineering has emerged, though, as a powerful approach to model and study various cardiac diseases in a controlled and physiologically relevant milieu. By combining cells, biomaterials, and engineering techniques, researchers have developed sophisticated tissue-engineered models that accurately recapitulate cardiac diseases, as, for example, MI, cardiac fibrosis, and hypertrophy.

The most common strategy relies on creating *in vitro* 3D tissue constructs based on CMs, along with other relevant cell types, such as ECs and CFs, especially derived from hiPS differentiation. In an approach very similar to the one used to manufacture cardiac patches intended for implantation, cells are seeded onto a wide variety of biocompatible scaffolds, as hydrogels of collagen and fibrin or PLGA nanofibers (Khan et al., 2015; Ronaldson-Bouchard et al., 2018; Zhao et al., 2019), providing structural support and guiding cell orientation and maturation within the construct. 3D bioprinting can also be used to obtain a specific distribution of ECM and cell elements (Khoury et al., 2021). As essential requirements, the resulting tissue-engineered cardiac constructs should exhibit physiological-like features, including aligned CMs, cell-cell connections, and tissue-level functionality, for proper modeling also of pathophysiological conditions.

Alternatively, biomaterials such as ECM-coated silicone/polydimethylsiloxane or thermo-responsive dishes are used as cell growth surfaces allowing CMs to adhere. Two types of models can be created thereafter, i.e., muscular thin films (Nishimura et al., 2004; Feinberg et al., 2007; Feinberg et al., 2012; Balashov et al., 2020) and cell sheets (Takeda et al., 2018; Ohya et al., 2021; Sasaki et al., 2021). Both can be considered cell multilayers, in which CMs might secrete their own ECM and form intercellular junctions at the basis of the syncytium activity. The main differences between the two models reside in the offered miniaturization possibilities. Differently from cell sheets, muscular thin films can be inserted in heart-on-a-chip and submitted to automated microperfusion (Nunes et al., 2013; Parsa et al., 2017; Veldhuizen et al., 2022). These microfluidic platforms require a relatively low number of cells (about 5.000 CMs), an incredibly reduced volume of the growth medium, and, consequently, of stimuli, e.g., growth factors, pathological agents, and so on. In disease modeling, this is particularly advantageous by contemplating a robust and repetitive investigation methodology characterized by an increased level of control over biological variables and costs; however, these platforms are not immune from shortcomings, as the need for specialized manufacturing facilities and technical difficulties to maintain stable operative conditions, especially for the generation of air bubbles.

While cell-free tissue engineering cannot be an option in cardiac disease modeling, the scaffold-free modality might be a valid alternative to biomaterials/cells-based, bioengineered tissues. Cardiac organoids or cardioids are created by self-organization or directed assembly of pluripotent stem cells undergoing both cardiac commitment and specification naturally occurring during heart organogenesis (Drakhlis et al., 2021; Hofbauer et al., 2021; Lewis-Israeli et al., 2021). Contrarily to other cardiac tissue-engineered models where the cell selection is operated by the researcher, these organoids ideally contain in nearly 100 µm in size all the cell types naturally observed in the heart, also with a chambered organization (Ho et al., 2022). Thus, they possess a stronger ability to replicate the full organ physiology (Kerr et al., 2021). Together with heart-on-a-chip, they can provide the most comprehensive readout. As observed previously, while the field of 3D cardiac tissue engineering is in continuous evolution, challenges remain, such as improving the maturity and functionality of bioengineered tissue disease models (still low are the cases in which evident T-tubules and M-bands, consistent H-zones and I-bands, and robust electromechanical coupling are demonstrated (Shadrin et al., 2017)), enhancing their scalability, and achieving long-term survival.

In the following subsections, some examples of cardiac disease modeling through myocardial tissue engineering will be described in the context of MI, cardiac fibrosis, cardiac hypertrophy, and cardiotoxicity (Supplementary Table 1).

##### 3.2.1.2 MI

MI (Tsao et al., 2023) is a well-studied pathology in the *in vitro* disease modeling mediated by cardiac tissue engineering. MI bioengineered models allow for the investigation of cellular responses, remodeling processes, and the effects of therapeutic interventions post-ischemia. In addition, they have been instrumental in studying aspects such as cardiac remodeling, inflammation, angiogenesis, and the influence of mechanical forces on tissue repair. Daly et al. generated a MI-like, human scar tissue model by bioprinting hiPS-CMs and CFs in a spatially controlled manner and were able to recapitulate the reduction of contractility and the substrate for arrhythmogenicity (Daly et al., 2021). Chin et al. investigated the relationship between cardiac cell volume adaptation and cell interactions. In particular, they generated cardiac spheroids with H9C2 myoblasts and GelMA hydrogels. These cardiac spheroids showed a limited ability of cellular volume adaptation to increasing substrate stiffness. In stiffer conditions, they replied with an overexpression of mechanomarkers, such as YAP, MRTF-A, and Lamin-a, and a nuclear size reduction (Chin et al., 2022). An *in vitro* 3D model of the post-MI tissue was advanced by Richards et al. by using cardiac organoid technology, oxygen gradients, and noradrenaline stimulation to mimic the ischemic settings of the damaged myocardium and the compensatory activity exerted by the nervous system for cardiac output recovery. This model was able to replicate the events occurring in the infarcted myocardium as the typical alterations in cell survival, metabolism, collagen synthesis, and calcium handling (Richards et al., 2020).

Models only reproducing oxidative stress help focus on the effects of the lowering oxygen tension, over-production of O_2_^·-^, NO·, NO_3_^−^, and generally reactive oxygen species (ROS) on CMs of the infarcted and border regions. Acun and Zorlutuna created a cardiac engineered tissue incorporating rat neonatal CMs and ECs derived from hiPS differentiation (iECs) or HUVECs in RGD-containing hydrogels. After H_2_O_2_ administration, they investigated HIF-1 alpha signaling as a player in oxidative stress, by knocking it down in iECs and HUVECs with small hairpin RNA. H_2_O_2_-treated CMs cultured in indirect (conditioned media) or direct contact with ECs submitted to RNA targeting showed reduced survival and increased apoptosis in both 2D and 3D cultures, thus suggesting HIF-1 alpha as an essential protective player in cell resistance to oxidative stress. In addition, they demonstrated that paracrine, as well as autocrine signals released by both ECs and CMs, and RGD, a tripeptide present in fibronectin, are involved in CM survival under oxidative damage, hence identifying a process dependence on both cell-cell and cell-ECM interactions. Transcriptomic levels of HIF1A-AS1, mitochondrial gene ROMO-1, and TGF-beta 3 were altered in cells after oxidative stress. The researchers showed that HIF1A-AS1 downregulation in ECs could partly increase CM viability, although they did not investigate which other factor could contribute to maximizing this effect in their 2D and 3D systems (Acun and Zorlutuna, 2017). The same group demonstrated in the context of a photocrosslinkable RGD-hydrogel that iECs stabilize CM survival under oxidative stress conditions by acting on mitochondrial CM pathways, involving drug metabolism-cytochrome P450, Rap1, and adrenergic signaling (Yue et al., 2017). Ischemia/reperfusion injury is responsible for CM death but also reduced contractility and loss of function. Sebastiao et al. developed a human ischemia/reperfusion model by submitting 3D hiPS-CM aggregates cultured in stirred tank bioreactors to different oxygen gradients. This model was demonstrated to recapitulate the phenotypic variations induced by ischemia/reperfusion on CMs, such as loss of viability, cell disarray, and secretion of specific pro-angiogenic and pro-inflammatory cytokines (Sebastião et al., 2020). Sharma et al. proposed another methodology to study ischemia/reperfusion damage in murine and human cardiac spheroids by varying oxygen concentrations between normoxic, hypoxic, and reoxygenation settings. With their innovative approach based on hanging drop of mouse cardiac cells or hiPS-CMs plus coronary ECs and CFs, they were able to confirm the lower resistance of CMs to ischemia in comparison to ECs and CFs populating the spheroids (Sharma et al., 2022a; Sharma et al., 2022b). By means of a cardiac ischemia on-a-chip model with 3D, anisotropic collagen hydrogels, hiPS-derived CMs, and CFs, Velduizen et al. studied the effects of different regimes of ischemia, as well as acute and chronic reperfusion and reoxygenation. Hypoxia did not induce any change in CM alignment, but a decreased synchronicity was observed. Hypoxia-responsive genes were generally upregulated. In particular, alpha-smooth muscle actin was overexpressed in CFs, revealing the onset of a strong fibrotic response to the ischemic stimulus. On the contrary, genes related to CM contractility resulted to be downregulated in their expression, thus recapitulating all the events typically occurring after an ischemic insult (Veldhuizen et al., 2022).

Oxidative stress models can also shed light on the possible causes that brought MSC therapy to inadequate performance or failure in acute and chronic myocardial infarction trials. Choe et al. encapsulated MSCs in an alginate hydrogel incorporating graphene oxide. This latter shows several features, by being water-dispersible, biocompatible, and free radicals-scavenger. H_2_O_2_ challenge did not significantly compromise cell viability in graphene oxide/alginate-encapsulated MSCs alone, as well as in co-cultured CMs. In particular, the latter showed increased survival and maturation with respect to its culturing in the presence of non-encapsulated MSCs (Choe et al., 2019).

With a similar aim to dissect the roots through which MSCs alone showed a trivial restorative effect in MI clinical cell therapies, Sylvester et al. focused on the effects of a rigid milieu and generated biopeptide-enriched poly(ethylene glycol)-acrylate hydrogels at different concentrations and stiffnesses used to encapsulate rat MSCs. Encapsulation at higher stiffness, used to simulate the scar environment, strongly reduced the ability of MSCs to express cardiac troponin T and growth factor release. These studies revealed that the low regeneration capability shown by these cells infused alone in the first clinical cell trials was strictly dependent on the adverse MI microenvironment (Sylvester et al., 2021).

##### 3.2.1.3 Cardiac fibrosis

Cardiac fibrosis, characterized by excessive collagen deposition and scar formation, is another pathological condition that has been effectively modeled using tissue engineering approaches. It can be secondary to many other cardiac diseases, including MI by aggravating global heart conditions. Thereby, its modeling is particularly relevant, also because it lends itself to testing targeted pharmacological treatments. By introducing fibroblasts or inducing fibrotic processes within the constructs, researchers have been able to mimic the outcome of dysregulated activation of resident CFs and, hence, the fibrotic microenvironment seen in diseased hearts. Therefore, these models have provided insights into fibrotic mechanisms, cell-cell interactions, and the effects of anti-fibrotic therapies.

With the purpose of studying both cardiac and lung fibrosis, Akinbote et al. developed 3D vascularized models based on CFs, hiPS-derived ECs, and fibrin. Two TGF-β stimulation regimens, different in concentration and time exposure, were applied. The researchers observed alterations in the microvasculature, permeability, and extravascular diffusivity exerted by CFs. Moreover, TGF-β treatments increased stiffness, alpha-smooth muscle actin expression, and release of metalloproteinases 1 and 9, although no clear effect could be observed in terms of collagen synthesis (Akinbote et al., 2021). Using GelMA, human fetal CFs, and hiPS-derived CMs, Bracco Gartner et al. also investigated fibrotic responses to TGF-β1. After 14 days, alpha-smooth muscle actin, periostin, and collagen types 1 and 3 resulted to be highly expressed at both genetic and protein levels. They tested the anti-fibrotic drug pirfenidone, which, however, did not reduce any transcript, apart from that of periostin, while affecting the synthesis of periostin, collagen 1, and metalloproteinase 2 proteins (Bracco Gartner et al., 2022). Cellular responses to TGF-β1 were also investigated by Ragazzini et al. in a cardiac fibrosis model based on stiffness-tunable GelMA and human cardiosphere-derived stromal cells. In particular, they explored the mechanistic relationship between TGF-β1 and YAP and its ability to compact the ECM. Moreover, they demonstrated that is sufficient to antagonize YAP with Verteporfin to reduce fibrosis. This drug is a selective inhibitor for YAP and has also received approval from the FDA for the treatment of age-related macular degeneration (Wei and Li, 2020). Verteporfin administration in this cardiac fibrosis model *in vitro* suppressed myofibroblast activation, excessive synthesis of ECM, and its remodeling (Ragazzini et al., 2022). Another approach of disease modeling recapitulated the ECM environment reminiscent of the pathological features observed at different stages of development. Spedicati et al. aimed to simulate by 3D engineering the early-stage fibrotic tissue developing in the immediate post-MI period. For 3 weeks, they cultured CFs upon 150 µm square-meshed scaffolds fabricated from polycaprolactone and gelatin by mussel-inspired adhesive surface pre-coating. A transition to myofibroblast phenotype was observed for CFs and excessive synthesis of ECM was shown with deposits bridging scaffold pores. Interestingly, using atrial CFs, the researchers could recapitulate atrial fibrosis (Spedicati et al., 2022). Also Zhu et al. investigated the effects of material rigidity on the phenotype of CFs in a biomechanopharmacology approach. They generated three types of rat models of cardiac engineered tissues (polyacrylamide gels and neonatal CFs) differing in the degree of stiffness (from nearly 29 to 125 kPa) and they observed a direct relationship between rigidity and cell phenotypic change. As in a murine model *in vivo*, the angiotensin-II receptor inhibitor candesartan was demonstrated to reduce proliferation, migration, and oxidative stress in CFs, by specifically lowering the protein levels of phosphorylated focal adhesion kinase protein (FAK), a cytoplasmatic, non-receptor tyrosine kinase, found to be overexpressed also in many tumors (Zhang et al., 2022b). Downstream to FAK, phosphatidylinositol 3-kinase/protein kinase B (PI3K/Akt), ERK1/2, NOX2, and ROS are components of the signaling cascade. The researchers elucidated, therefore, the important role of this protein activation in orchestrating cardiac fibrosis for both pro-fibrotic and oxidative stress tissue responses, as they also proved challenging the cardiac engineered tissues with the specific FAK inhibitor PF-573228 (Zhu et al., 2023).

CF phenotypic switch was obtained by Santos et al. by molding and tuning the geometrical properties of a scaffold of collagen mixture. Playing with the uniformity level of the engineered constructs, they observed the onset of the fibrotic pattern in uniform cardiac tissue characterized by higher stiffness, poor elasticity, and reduced contractility. In these modeling approaches, the molding was found to be the principal player in cardiac fibrosis recapitulation over other parameters, as the material substrate, its stiffness degree, or the presence of fibrotic signals (such as, pro-fibrotic TGFβ1 and ROCK-actin-LOX cascade inhibition). A proof-of-validity of this disease modeling was obtained by replacing healthy CFs with cells derived from patients with ischemic or dilated cardiomyopathy. The CFs isolated from the heart of a patient with a hypertensive form of dilated cardiomyopathy showed an increased inability to contract, while stiffer and less extensible engineered tissues were generated with all pathological cell types in uniform settings (Santos et al., 2022). With a bioinspired molding modality, Liu et al. reproduced antenna-like trichomes with 3D bioprinting and manipulated cell-laden microgels passing through microscale droplets. Rat CFs encapsulated by collagen were inserted in these microgels with catcher-like structures. Through this, the researchers recreated the milieu of cardiac fibrosis after stimulation with TGFβ1, by utilizing a multiwell, catcher-like platform that has the potential to facilitate the simultaneous testing of several drugs for high throughput readout (Liu et al., 2021b). More recently, Waldrop et al. investigated the role of hemodynamic stress on cardiac remodeling. To this aim, they focused on the effects of volume overload on cardiac engineered tissues composed of rat cardiac myoblasts (H9C2 cell line), healthy adult human dermal fibroblasts, and hydrogel. By exposing these tissues to 10 mmHg pressure at 1 Hz for 4 days, not only CFs replied undergoing a myofibroblast switch, but also collagen type 1, TGFB1, and CCR2 resulted to be upregulated, thus recapitulating the typical hallmarks of cardiac fibrosis, included inflammation and oxidative stress. Analogous observations related to this *in vitro* hybrid-species system were collected by the same authors from an *in vivo* murine model of arteriovenous fistula (Waldrop et al., 2023).

##### 3.2.1.4 Cardiac hypertrophy

Tissue-engineered models have been utilized also to study cardiac hypertrophy, a condition characterized by an increase in CM size and alterations in contractile properties. By subjecting the engineered constructs to hypertrophic stimuli such as mechanical stretch or neurohumoral factors, researchers can mimic the hypertrophic response observed in diseased hearts. These models have shed light on the signaling pathways involved in hypertrophy, and contractile dysfunction, as well as the effects of therapeutic interventions.

One of the first approaches to *in vitro* modeling of this disease focused on determining the cellular effects of volume overload in a microfluidic platform. Parsa et al. fabricated multilayer arrays of microbioreactors, in which non-contacting, multiple layers of tissue culture and pneumatically actuated control were incorporated into the platform with real-time microscopy monitoring. Each cultured tissue was 580 µm in thickness and integrated 500 cells (rat neonatal CMs and CFs) and collagen gels. After a gradient of mechanical stress, these engineered tissues overexpressed in a strain level-dependent manner atrial natriuretic peptide, β-myosin heavy chain, and skeletal α-actin genes (Parsa et al., 2017). Hence, cells replied to the mechanical stimulus by reactivation of the so-called fetal gene program, which is expressed in the heart during embryological development and is switched off in adult life in physiological conditions. A polygenic cardiac disease microchip model was generated by Zhao et al. by growing a cardiac engineered tissue in collagen/Matrigel hydrogel, human CFs, and hES-and hiPS-CMs (either atrial or ventricular). Electrical conditioning was applied in a stimulation chamber after 1 week from the creation of the cardiac engineered tissue resulting in an expression pattern consistent with the one of the adult heart and the typical, distinctive responses of the atrial and ventricular myocardium. The administration of drugs with chamber-specific cardiac effects, such as the L-type Ca^2+^ channel blocker nifedipine, the sarco/endoplasmic reticulum calcium ATPase inhibitor thapsigargin, and the type 2 muscarinic receptor agonist proved the effective engineering of atrial and ventricular myocardial tissues. In addition, a comparison between engineered ventricular myocardium generated with cells derived from patients affected by primary hypertension and from healthy subjects further confirmed the suitability of this *in vitro* system to recapitulate the signs of left ventricular hypertrophy. In these conditions, the pathways related to cardiac enlargement, cardiac dilatation, cardiac dysfunction, heart failure, and cardiac hypertrophy signaling were activated, by recapitulating the adverse cardiac remodeling observed clinically (Zhao et al., 2019). Micro-heart muscle arrays with sarcomeric assembly and tissue alignment were generated by Guo et al. by seeding hiPS-CMs on a fibronectin-decorated elastomer using a mold with a dog bone shape. By varying the elastomer stiffness in a physiologic range (5–30 kPa), a higher contractility frequency was observed in conditions simulating afterload. This paralleled an increase in calcium influx, but a reduction in developed traction force. Cells showed also morphological changes by a slight rise in size, thus reproducing some of the key cellular features of cardiac hypertrophy (Guo et al., 2021b).

##### 3.2.1.5 Cardiotoxicity

By culturing the tissue constructs in microfluidic platforms, researchers can mimic physiological blood flow, nutrient supply, and drug distribution. This enables the evaluation of drug efficacy and toxicity in a more relevant *in vitro* environment. These models have demonstrated the ability to predict drug responses more accurately than more traditional approaches and, thus, hold promise for personalized medicine applications. Established mammal models, mainly transgenic mice, were largely utilized to test novel pharmacological therapeutical hypotheses. Potential heart toxicity was often undiscovered in the experimental animal and manifested lately during the clinical application, causing safety issues and drug withdrawal from the market. This relatively poor predictive ability depends once again on the molecular and electrophysiological differences between experimental animals and humans. *In vitro* traditional approaches could show other weaknesses, as the consideration of a unique ion channel or the use of non-human cells. Among 2D cell culture systems, hiPS-CMs have been already demonstrated as suitable tools to evaluate the cardiotoxic effects of many drugs, and specific guidelines, such as the ones promoted by the Comprehensive *In Vitro* Pro-arrhythmia Assay (CiPA) and the Japan iPS Cardiac Safety Assessment (JiCSA) initiatives, have been released to standardize *in vitro* drug testing and enhance the translational value of collected outcomes (Ando et al., 2017; Blinova et al., 2017; Kanda et al., 2018; Takasuna et al., 2019; Kanda et al., 2021).

Pro-arrhythmic toxicity can be provoked by drugs with direct effects on the heart but can also be the adverse effect of pharmacological therapies administered for other body/disease targets. By means of nearly 2.000 hiPS-CMs, Huebsch et al. generated micro-heart muscle arrays with uniaxial alignment and contractility, as well as sarcomeric assembly, which demonstrated reproducible, physiological responses to drugs as β-adrenergic agonist isoproterenol and calcium antagonist verapamil in a proof-of-concept (Huebsch et al., 2016). The ability to induce the lethal condition of torsade de pointes was assessed in several cardiac bioengineered models. A heterologous engineered cardiac sheet, composed of CMs and non-cardiac cells derived from hiPS differentiation, was produced to simulate the substrate of this lethal arrhythmia condition. Administration of the HERG K^+^ channel blocker E-4031 provoked a sustained tachyarrhythmia with spiral wave reentry waveforms, the classical signs observed in patients affected by torsade de pointes. Interestingly, 3D architecture and cellular heterogenicity in cell sheet fabrication identified necessary and sufficient design conditions to induce this pathological behavior in engineered cardiac tissues (Kawatou et al., 2017). The gastrokinetic agent cisapride is recognized to cause fatal arrhythmias and long QT syndrome when overdosed, by inhibiting hERG potassium voltage (Kv)-gated channels (in particular, KCNH2 and KCNH6 channels) (Lu et al., 2022). Tsui et al. modeled the proarrhythmic effects of cisapride in terms of action potential prolongation and beat interval abnormalities making use of hybrid hydrogels, composed of decellularized porcine myocardium, enriched with reduced graphene oxide, and seeded with hiPS-CMs. Also in this case, it emerged the pivotal role of the 3D scaffold to improve CM maturity and recapitulate cellular pathophysiologic switch in response to drug administration (Tsui et al., 2021). Several environmental toxicants and industrial chemicals, such as bisphenol A, are proarrhythmic; however, little is known about the action mechanism, through which they induce heart rhythm disturbances. Kofron et al. tested the effects of bisphenol A on cardiac engineered tissues generated with a cell mixture of hiPS-CMs and human CFs forced to form spheres in non-adhesive agarose gels and validated with high- and low-risk hERG channel blockers (such as, E4031 and ranolazine). Lactate purification and a 5% ratio of CFs in these self-assembled microtissues were found to be indispensable to improve contractility. The exposure of these microtissues to the environmental pollutant bisphenol A activated a complex, biphasic response depending also on its dosage. By increasing the pollutant concentration, they observed as first hERG channel blocking and, lately, alterations of the inward rectifier current. By complementing these results with an *in silico* model integrating experimental outcomes generated with other drugs, the researchers were able to formulate a mechanistic hypothesis on the action of the pollutant. Following this latter, in subjects exposed to bisphenol A, the myocardial tissue might reply with a compensatory effect to the immediate potassium channel blockade and, as a combinatorial consequence, the final result is the shortening of the action potential duration (Kofron et al., 2021). Archer et al. considered a large panel of FDA-approved cardiotoxins (a total of 29) belonging to anti-arrhythmic, neoplastic, fungal, and psychotic drug classes. Their administration might lead to structural and non-structural cardiac effects and is clinically associated with the onset of MI, myocarditis and/or cardiomyopathy, and finally chronic heart failure. All tested drugs were found to alter the physiological electrical behavior of engineered cardiac spheroids (hiPS-CMs, primary human cardiac microvascular ECs, and primary human CFs). Depending on the typology and level of exposure, rhythm alterations could be observed as a result of the effects on different primary or secondary cellular targets, such as cell viability and the organelles mostly involved in cardiac tissue metabolism. By assessing mitochondrial membrane potential, ER integrity, ATP depletion, and cell release of cardiac biomarkers (i.e., cTnI, CK-MB, and FABP-3), the researchers were able to generate a mechanistic fingerprint for each cardiotoxin, not necessarily linked to the class it belongs and/or to CM-selective stress. As an example, doxorubicin exposure (30 μM, 72 h) was found to induce a higher release of FABP-3 with respect to other biomarkers, as well as a simultaneous, overall impairment in ATP levels, ER integrity, and mitochondrial membrane potential (Archer et al., 2018). This anthracycline is widely employed clinically for the successful treatment of several malignancies, including breast cancers and non-Hodgkin lymphomas (Dempke et al., 2023). Its use must be particularly cautious in subjects at high cardiovascular risk being associated with heart failure. Therefore, there is a great interest in exploring the mechanisms of its side effects and developing administration modalities that can reduce them. By using human cardiac organoids, Richards et al. demonstrated the unprecedented feasibility to test the effect of doxorubicin in infarcted hearts in physiologically relevant settings. In hypoxia-stimulated cardiac organoids, doxorubicin contributed to inducing a further decrease in cell viability and contractility. An antineoplastic concentration of 1 µM was sufficient to reduce heart beating frequency to zero, while the same effect could be reached in control organoids not before the 50 µM drug challenge. Concomitant features such as increased apoptosis, sarcomeric disarray, and CF outgrowth could be detected by recapitulating the loss in contractility (reduced LVEF) and cardiac fibrosis observed in doxorubicin-treated cardiopatic patients (Richards et al., 2020). In a similar scenario of pathological oxygen tension and doxorubicin treatment in a cardiac spheroid model, Sharma et al. focused on the mechanistic events following ischemia/reperfusion damage, and drug toxicity by deeply studying the molecular alterations in terms of the sarcomeric array, calcium handling, cell viability, and cardiac remodeling (Sharma et al., 2022a). In a 3D bioprinted cardiac spheroidal droplet-based system, El Khoury et al. confirmed the side effects in cell viability and contractility at 1 µM and proved that the administration in tissue culture of the strong antioxidants *N*-acetylcysteine and Tiron could alleviate cardiotoxicity through the cardioprotection mediated by apoptotic caspase-3 inhibition (El Khoury et al., 2023).

## 4 Unmet needs and issues to be addressed for clinical translation

Cardiac tissue engineering holds immense relevance in surgery, disease modeling, and pharmacological testing. It has the potential to improve patients’ outcomes in cardiac surgery, provide a better understanding and treatment of cardiac diseases, and facilitate more accurate and efficient drug discovery and development processes. As the field continues to advance, it is expected to have a significant impact on cardiovascular medicine and pave the way for innovative therapeutic strategies. However, many technological issues remain to be addressed. In addition, applications need to be broadened in order to offer valid solutions, either surgical or pharmacological, to all patients affected by cardiovascular diseases.

Heart regenerative medicine continues to advance in its three main categories, as cell-based treatments, cell-free scaffold- or cellularized scaffold-based cardiac tissue engineering. Despite significant progress in each of these approaches, their clinical translation has been severely hampered by two major barriers to successful structural and functional replacement of the damaged myocardium: poor engraftment of engineered tissue into the damaged cardiac muscle and weak electromechanical coupling of transplanted cells with the native tissue. Beyond undeniable therapeutical advantages related to each approach over standard treatments, several specific issues can be still identified, thus needing further effort for their ultimate translation into the clinic.

Although cell-free scaffold technology has achieved long-term advances in the field of tissue engineering and exciting results in animal testing [for instance, the cardiac patch devised by Shah et al. (2019)], several persistent challenges remain. For optimal grafting and preservation of the myocardial engineered tissue, optimization of mechanical support of the scaffold, facilitation of cell migration and angiogenesis, and partial preservation of the heart muscle cells within the lesion and the area beneath the patch must be pursued (Serpooshan et al., 2013). In fact, the tissue-specific mechanical environment, in which an acellular scaffold is employed, plays a role in determining its surgical success. The selection of the ideal mechanical properties, such as stiffness or elasticity (Young’s modulus), requires more investigation. For example, additional work should be realized to understand whether an elastic acellular scaffold, which may better approximate the native mechanical properties of the heart, is more favorable than a more rigid one that, contrariwise, may provide greater structural support (Pattar et al., 2019). The goal is to leverage acellular scaffold technology to potentiate the activation and drive endogenous mechanisms of repair and repopulation (angiogenesis or vasculogenesis included), while attenuating the activation of cardiac myofibroblasts. In doing so, the maladaptive cardiac structural remodeling that ensues from ischemic injury to the heart might be managed more efficaciously. A first starting point could be represented by standardizing scaffold production protocols (e.g., decellularization, 3D bioprinting, *etc.*) and characterization methods, in order to obtain ideal biological and mechanical properties. Moreover, effective sterilization methods should be developed, especially those for virus eradication, while minimizing the impact on the mechanical and biological characteristics of scaffold materials. Furthermore, functional strategies for thrombotic and inflammatory response reduction should be implemented for successful tissue and organ regeneration. Currently, thrombosis remains a challenge when whole-heart dECMs are used *in vivo*, as recently emphasized by Taylor et al. (2018). Finally, the ideal decellularization/production protocol should avert any risk of immunogenicity. Indeed, the threat of immediate- or late-onset rejection still exists in humans when using animal sources. The assessment of allo- or xeno-immunogenicity and biocompatibility is critical for final health authority approval, commercial viability, and clinical application. Immunogenic risks have been long explored for heart valve and blood vessel replacements (Iop et al., 2009; Naso et al., 2013; Cooper and Pierson, 2023), but still deserve similar attention for myocardial engineered tissues originating from animal scaffolds.

Several setbacks can also be identified in the significantly advanced, cell-based, scaffold-free cardiac therapy approaches. First, there is no consensus over the optimal cell type or delivery method to be used since the outcome could be widely influenced also by other variables, as MI stage, delivery route, *etc.* (Iop et al., 2018; Pagano et al., 2019). Additionally, there is a lack of control over the differentiation fate of the stem/progenitor cells upon implantation. The immaturity of hPS-derived CMs remarkably limits their potential for clinical application (Pfannkuche et al., 2009; Kawamura et al., 2012). Cell sheet approaches have some drawbacks including that they are difficult to handle and relatively fragile, and potentially limited in thickness in multilayered settings (Roberts et al., 2019). Suboptimal retention and survival of stem cells in the ischemic myocardium after injection remain major obstacles limiting the efficacy of cell-based therapy (Bolli and Ghafghazi, 2017). Moreover, low graft survival and cell death can result from ischemia, apoptosis, loss of ECM, and inflammation. Indeed, the establishment of a continuous blood supply to cardiac cell sheets is necessary for successful engraftment.

Cellularized scaffolds partially solve engraftment problems thanks to the natural vasculature ECM scaffolding of acellular tissue and organs that can provide oxygen and nutrients to the whole thickness and, hence, guarantee cellular survival and function. However, establishing novel angiogenesis or vasculogenesis and electromechanical coupling in the graft site remains an issue. Previous generations of the cardiac patch strategy focused on improving stem cell retention and promoting heart tissue repair after MI (Annabi et al., 2013; Tang et al., 2017)]. Despite the progress, most recent versions still face the unmet task to promote both cardiomyogenesis and angiogenesis at the injured site after implantation (Ogle et al., 2016). Again, being the strategy based on cells, the maturation state of stem cells-derived CMs needs to be enhanced, preventing any teratogenicity and immunological incompatibility in the host. In addition, while cell therapy could exploit injection technique as the route of delivery, there is a need to elucidate the best modality of bioengineered myocardial patch deployment to ensure optimal engraftment at the interest site.

**FIGURE 4:**
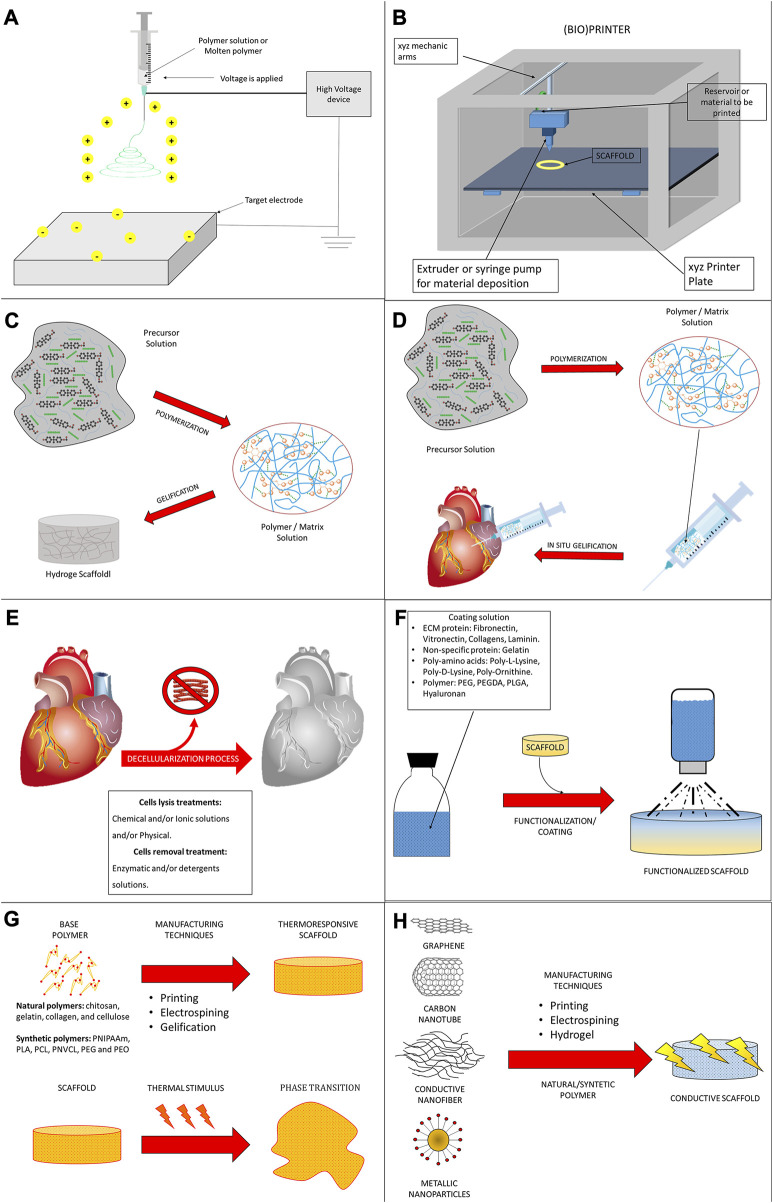
Current modalities of scaffold engineering. **(A)** Electrospinning uses electric force to draw charged threads of polymer solutions or melts up to fiber diameters in the order of hundred nanometers. **(B)** 3D (bio)printing is the construction of a 3D scaffold from a digital 3D model. The chosen material is deposited by an extruder or syringe needle, joined, and solidified typically in layer-by-layer way. **(C)** A hydrogel scaffold is a biphasic material, a mixture of porous permeable solids and insoluble water, characterized by insoluble 3D network of natural or synthetic polymers with an intrinsic hydrophilic character due to their functional groups. **(D)** An injectable hydrogel is generally based on the possibility of some biomaterials to be injected as liquid into the human body, and then, *in situ* transformed into solid gels. Generally, a mixture of the polymer/monomer solution (called precursor) and therapeutic agents in the syringe is feasibly administrated to a desired site in the body since its viscosity is low enough to be injected through a syringe needle. Then, the therapeutic agent-loaded hydrogel is formed by crosslinking reaction where its viscosity drastically increases during the sol-to-gel transition phase. The gelation typically occurs by forming the crosslinks via chemical or physical reactions. **(E)** Decellularization is a procedure that removes cells and non-ECM proteins from a native tissue using physical, chemicals and/or enzymatic treatments. In opportune conditions, decellularized scaffolds preserve the original ECM of tissues and organs, including the network supporting vasculature and innervation. **(F)** Functionalization is a process aiming at adding new features, capabilities, or properties to a material by changing its surface chemistry. It is performed by decorating the material surface with molecules or nanoparticles, either with a chemical bond or just through adsorption. **(G)** Temperature-responsive polymers are a class of “smart” materials that exhibit drastic and discontinuous changes in their physical properties in response to temperature. Thanks to this property, thermoresponsive materials find application into drug delivery, gene delivery, and *in situ* tissue engineering. **(H)** Electroconductive materials have the capacity to carry electric current, thus conveying bioelectronic signals. Artificial electroconductive materials, as conductive peptides (composites containing gold or silver nanoparticles and nanowires), carbon-based materials (like graphene and carbon nanotubes), and synthetic conductive polymers (originating from the field of organic electronics) can be mixed with different synthetic/natural polymers and used to realize electroconductive scaffolds.

Beyond the specific limitations related to each of the three main strategies, cardiac regenerative medicine faces general, multiple problems, including significantly poor cellular survival, modest electrophysiological coupling, and a lack of integration/engraftment of designed tissues with the host myocardium. As cardiac regenerative medicine studies continue onward, it should not be overlooked the primary goal, i.e., the generation of bioengineered equivalents with the capability to provide functional myocardial muscle replacement. Therefore, tissue engineering methodologies should incorporate structural, mechanical, and electrical cues in scaffolds, biological and chemical signals in purely cellular sheets, and a blend of them in hybrid products. To that aim, novel regenerative medicine techniques based on stem cell bioengineering and micro- and nanoscale technologies (Figure 4) are currently under development to overcome these important deficiencies.

Myocardial tissue engineering is making progress also in pathology research. Over traditional modeling approaches based on heterologous systems, experimental animals, and 2D cell cultures, 3D hiPS-based models are increasing the translational relevance between preclinical research and clinical applications, offering a more performing platform for understanding disease mechanisms, testing therapeutics, and ultimately improving patients’ outcomes. Notwithstanding the promising results collected so far with the bioengineering of physiological and pathophysiological myocardial tissues in terms of mechanism study and drug testing, many efforts need still to be dedicated to generating genuine replicas of human cardiovascular diseases. Yet, few models are available to study pathologies with atria-, conduction tissues-, or ventricles-specific implications (Lemme et al., 2018; Zhao et al., 2019; Kerr et al., 2021; Williams et al., 2021; Krause et al., 2022). Moreover, there is nevertheless a paucity of *in vitro* platforms of innervated cardiac muscle (Whye et al., 2022), which could be essential to study the diseases, in which the impairment of the autonomic nervous system has tremendous effects on heart function, as in the case of Alzheimer syndrome and other neurodegenerative diseases (Elia and Fossati, 2023). The progressive development of specific protocols to enrich CM subpopulations during hiPS differentiation (Cyganek et al., 2018; Kerr et al., 2021) will not only solve the issues of graft unfunctional behavior described before but will further help to generate chamber-specific pathology models, also for rare congenital diseases. Moreover, it will allow to test in a relevant pathophysiological environment novel drug hypothesis, especially for diseases so far untreatable, as well as target specificity of pharmacological therapies. As for models aiming at studying cardiac fibrosis by opportunely mixing CMs and CFs, it would also be essential to establish the right ratio of CMs and nervous cells able to reproduce the physiological, innervated myocardial tissue. Among other diseases that remain little studied using cardiac engineered tissues are acquired pathologies, as the ones related to metabolism and infections. Interestingly, Lewis-Israeli developed a fetal heart model with cardiac organoids and tested the effects of hyperglycemia to simulate maternal diabetes and evaluate its impact on the cardiac health of the unborn child (Lewis-Israeli et al., 2021). By means of a heart-on-a-chip system, Wang et al. modeled *in vitro* the complex pathological dynamics of non-genetic cardiomyopathy induced by angiotensin II; in addition, they observed the pleiotropic cardioprotective superiority of relaxin treatment when compared to other drugs, as losartan and saracatinib (Wang et al., 2021). During the COVID-19 pandemic, it became clear the pathological involvement of heart function with the possible diagnosis of viral myocarditis in infected subjects. 2D hiPS-CM cultures were widely utilized to study the pathophysiology of the Sars-COV2-mediated viral disease. Recently, Bailey et al. established an engineered heart tissue model incorporating hiPS-CMs, fibroblasts, and/or macrophages, in which the inoculation of SARS-CoV-2 for 7 days successfully reproduced COVID-19 myocardial pathology. This model enabled to study the mechanisms of viral pathogenesis and the progressive impairment in contractility, cytokine release, sarcomeric organization, and cell viability (Bailey et al., 2021). With analogous methodology, models of other infective myocarditis, either with bacterial, fungal, or viral etiology, could be generated by the challenge of a bioengineered myocardial tissue replica with a specific pathogen. As for the recent alarm raised by the World Health Organization on future infectious threats (Imagining the future, 2022), such a modeling approach could have a strong translational impact for prompt understanding of the specific pathomechanism, identification of efficacious pharmacological treatments, and mitigation of the global sociomedical impact of a given microbial biohazard. For such a reason, it would also be necessary to integrate immune cells, as, for example, macrophages, in order to reproduce the full complexity of the physiological cardiac tissue endowed with a resident population of cells deputed to defense (Swirski and Nahrendorf, 2018; Banerjee et al., 2023).

Apart from metabolic and infective forms, cardiovascular diseases might onset with aging, which is a well-recognized agent of progressive and irreversible deterioration of biological processes and, hence, body function. The pathological effects of aging could be well studied with cardiac engineered tissue models, as shown recently by two independent groups using heart-on-a-chip platforms (Acun et al., 2019; Budhathoki et al., 2022). Despite the physiological differences between women and men being recognized, gender medicine is an insufficiently explored branch of Modern Medicine, and treatments remain generally established for a male subject with around 70 kg of weight. Concerning heart biology and physiology, too, gender differences related to cardiovascular diseases are known and increasingly evidenced, but little considered to personalize healthcare management (reckelhoff, 2014). As recently emphasized by Lock et al. (2022), sex-specific, engineered cardiac tissues incorporating hiPS-CMs derived from female or male subjects/patients could appropriately fit as suitable research tools in the frame of gender cardiovascular medicine to better investigate metabolic differences (specific endocrine stimulation, cellular behavior, *etc.*) and tailor pharmacological therapies. Similarly, these models could serve to better analyze the propensity of some geographical or ethnic groups or populations to develop specific cardiovascular diseases with genetic or environmental etiologies (Teshale et al., 2023).

In order to reach this level of modeling complexity, ancillary technologies and methodologies should also undergo considerable improvement. As aforementioned, CM functional maturation is still an issue when dealing with differentiated hiPS (or ES). Biochemical stimulation inspired by heart embryonic development is essential to induce mesoderm and contribute to cell specification of pluripotent stem cells, but cellular peculiarities of fully differentiated CMs, such as physiological assembly of sarcomeres, appropriate functioning of all ionic currents, or tissue alignment, are yet to be faithfully reproduced. The use of biomaterials with pro-alignment or electro-inductive physical cues, or possessing tissue-specific matrikine signals can strongly contribute to pushing hiPS differentiation over the stage of the embryonic CMs (Chun et al., 2015; Khan et al., 2015; Zhang et al., 2017a; Ren et al., 2017), but might be inadequate to reach full maturation. Mechanical or electrical stimulation could be a further tool to boost hiPS-CM differentiation (Kolanowski et al., 2020).

Along with the increasing efforts to fully differentiate CMs from pluripotent stem cells and, hence, derive functional engineered cardiac tissues, research is also focusing on designing and developing devices that can monitor in real-time and thus allow to progressively follow and reconstruct the whole tissue dynamics, also in response to exogenous stimuli. Technical challenges exist in the full thickness analysis but several researchers have developed advanced stimulating/sensing methods and equipment, e.g., microscopy visualization, contractility measurements, mechanical manipulation, and related high-throughput data collection that can easily combine with the engineered tissue models and, therefore, create automated, integrated platforms for robust readout (Ramade et al., 2014; Pointon et al., 2017; Sasaki et al., 2018; Gracioso Martins et al., 2021; Ohya et al., 2021; Sasaki et al., 2021; Oguntuyo et al., 2022). So far, a relatively short time of a few hours or days has been dedicated to observing drug-induced alterations. Relying on these sophisticated platforms could sustain the realization of long-term pharmacology testing aiming at verifying efficacy, calibrating dosage, and unraveling possible cardiotoxic effects related to a continuous administration of a given drug or chemical compound. Moreover, collected outcomes are useful to generate and/or validate *in silico* models, i.e., computational replicas of experimental observations (Nordsletten et al., 2021). This latter could, in turn, reduce the need for experimental work by increasing the predictive power related to the safety and efficacy of a pharmacological treatment hypothesis. Moreover, in combination with artificial intelligence, it could anticipate the discovery of novel targets and agents to be validated in pathophysiological engineered heart tissues.
