# Supplementary material for: Advances in the design, generation, and application of tissue-engineered myocardial equivalents
Source: Front Bioeng Biotechnol. 2023 Sep 22;11:1247572. doi: 10.3389/fbioe.2023.1247572 (PMC10559975; doi:10.3389/fbioe.2023.1247572)
Supplement: Supplementary file 1 [file Table1.docx]

# Tables

Table 1 Cells-based approaches in the generation of tissue-engineered myocardial equivalents

| **MAIN TECHNOLOGY** | **CELL**  **TYPE** | **SCAFFOLD TYPE** | **PRECLINICAL APPLICATION** | **CLINICAL APPLICATION** | **Ref.** |
| --- | --- | --- | --- | --- | --- |
|  |  |  |  |  |  |
| **IN VITRO ENGINEERED TISSUE WITH HYDROGELS OR SOLID MATRICES** | Human cardiospheres | Gelfoam or collagen (C-RGD) scaffolds | Both scaffolds were able to improve survival, as well as cardiac commitment and differentiation of cardiospheres-derived cells in vitro. | / | (62) |
|  | Rat cardiomyoblasts (H9C2) | Gelfoam, alone or combined with Matrigel and/or VEGF | MI hearts were submitted ex vivo to implantation with different engineered tissues and transplanted in vivo heterotopically. After 4 weeks, Gelfoam/Matrigel grafts demonstrated more viability and integration with host’s tissues. Heart performance was also ameliorated. | / | (63) |
|  | Human neonatal foreskin fibroblasts and hES-CMs | Hydrogels of medical-grade bovine collagen | Defined mixture of CMs and fibroblasts was proven essential in vitro to reach a viscoelastic stroma able to support cardiomyogenesis | / | (68) |
|  | hiPS-CMs | Low collagen hydrogel | In vitro engineered heart tissues assembled with high density of hiPS-CMs and low collagen hydrogel increased maturation in response to electrical stimulation and stretch. | / | (70) |
|  | Rat cardiomyoblasts (H9C2) | Fibrin patch loaded with neuregulin-1 | A natural growth trend was described for H9C2 cells in vitro seeded onto fibrin scaffolds enriched with neuregulin 1 with higher cell proliferation with respect to plain matrices. In a rat MI model in vivo, the loaded scaffolds reduced resident CMs’ apoptosis. | / | (78) |
|  | hES-CMs | Fibrin hydrogels | Fibrin hydrogels supported the growth and maturation of hES-CMs in vitro, generating functional engineered heart tissues able to show chronotropic responses and effectively reply to proarrhythmic drugs. | / | (85) |
|  | Neonatal rat ventricular CMs | Silk fibroin lamellar scaffold | Lamellar scaffolds in silk fibroin with varying silk proteins were seeded with neonatal rat CMs in a perfusion bioreactor. The protein components and their assembly influenced importantly the physical properties of the scaffolds, as the porosity and stiffness, and, consequently, the phenotype of seeded CMs. | / | (86) |
|  | Rat cardiomyoblasts (H9C2) | Polypropylene glycol, polyethylene glycol urethane, and selenide | Selenium-incorporating polymeric hydrogels did not alter the viability of H9C2 cells in vitro. When tested in an in vivo MI model in the rat, they showed ﻿anti-inflammatory and anti-fibrosis activities and ameliorated cardiac function. | / | (104) |
|  | Neonatal rat ventricular CMs | ﻿Cellulose nanocrystals | ﻿Several types of mussel-inspired adhesive ionic hydrogels were generated based on a redox-responsive technology and showed different properties in terms of ﻿morphological, conductive, and mechanical characteristics. When challenged in vitro with CMs in hypoxic environment, they showed anti-oxidation and anti-inflammatory effects. In vivo in a rat MI model, the hydrogel prompted tissue repair and function. | / | (105) |
|  | Neonatal rat ventricular CMs | Chitosan, vitamin C | So-obtained chitosan-vitamin C hydrogel did not modify cell viability in vitro, also in hypoxic conditions. | / | (110) |
|  | Rat cardiomyoblasts (H9C2), BM-MSCs | Aldehyde dextran sponge, resveratrol | ﻿The sponge loading anti-oxidative/autophagy-regulating resveratrol was tested for cytocompatibility in vitro with H9C2 cells and then applied in the rat heart via capillary removal of interfacial water from the tissue surface. In vivo effects of cardioprotection were observed. | / | (111) |
|  | hES-differentiated CPCs | Fibrin | / | Cardiac patches obtained in vitro with fibrin and hES-differentiated CPCs were evaluated in patients with severe ischemic dysfunction of the left ventricle. Survival at 1 year was disclosed for 4 out of 6 patients with amelioration of systolic motion and no arrhythmias. | (228) |
|  | Human umbilical cord MSCs | Collagen | / | Collagen hydrogels seeded with MSCs were implanted intramyocardially in patients with chronic heart failure in a randomized, double-blind clinical trial. The procedure was demonstrated safe and allowed to reduce the infarct size with respect to the sole cell infusion. | (229) |
|  | Worthon’s jelly-derived MSCs | Extracellular matrix (not specified) | / | 40 patients: ongoing recruitment | (230) |
|  |  |  |  |  |  |
| **Electroconductive and magnetic materials** | Neonatal rat ventricular CMs | Single walled carbon nanotubes incorporated in rat tail type I collagen hydrogels | Nanocomposite hydrogels seeded with neonatal rat ventricular CMs did not induce cytotoxicity and improved cell alignment. | / | (71) |
|  | Neonatal rat ventricular CMs | Carboxyl-functionalized multiwalled carbon nanotubes and rat tail type I collagen hydrogels | In vitro seeding of neonatal rat ventricular CMs into functionalized hydrogels improved cardiac cell function with respect to pure collagen matrices. | / | (72) |
|  | Rat cardiomyoblasts (H9C2) and hBM-MSCs | Chitosan-silk fibroin hydrogels engineered with gold nanoparticles | Gold nanoparticles in hydrogels did not modify the viability of H9C2 cells and hBM-MSCs and contributed to maintain CM phenotype, both in vitro and in vivo. | / | (80) |
|  | hiPS-CMs | Iron oxide magnetic nanoparticles and collagen hydrogel | Magnetic nanoparticles are incorporated in hiPS-CMs via antibody binding. In vitro application of magnetic field to hiPS-CMs seeded into collagen hydrogel improved cell alignment. In vivo grafting did not induce any alteration on heart performance in a rat model. | / | (73) |
|  | Neonatal rat CMs and hiPS-CMs | Collagen, alginate, and electroconductive poly(3,4-ethylenedioxythiophene):polystyrene sulfonate | Electroconductive biohybrid hydrogels were not cytotoxic, stabilized, and supported CM electrical behavior with both cell types in vitro. | / | (83) |
|  | Neonatal rat CMs | Hydrogels of polycaprolactone and silk fibroin engineered with carbon nanotubes | Aligned conductive nanofiber yarns network in hydrogels assisted CM alignment, elongation, and maturation in vitro. | / | (84) |
|  | Neonatal rat ventricular CMs | Gold nanowires and alginate | The engineering of alginate with gold nanowires increased the material conductivity and improve electrical communication between seeded CMs in vitro. | / | (94) |
|  | Neonatal rat ventricular CMs and rat aortic ECs | GelMA, PEGDA, MXene Ti_2_C and mussel-inspired dopamine | This mussel-inspired cryogel was able to increase CM alignment in vitro, by generating a synchronously beating myocardial engineered tissue. In addition, it demonstrated pro-vasculogenic features when implanted in a rat model of MI in vivo. | / | (96) |
|  | Mouse atrial HL1 CMs | Silicon carbide and silicon nanowires | These biocompatible elements were used to bridge CMs and demonstrated the ability to restore intercellular conductance in vitro with HL1 cells and in vivo in a rat model of heart cryoinjury. | / | (98) |
|  | Neonatal rat ventricular CMs | Polypyrrole nanoparticles, GelMa, mussel dopamine, and PEGDA | The obtained dopamine-based cryogel showed an excellent cytocompatibility and promoted cell differentiation when seeded with CMs in vitro, as well as favored cardiomyogenesis when implanted in vivo on rat infarcted myocardium. | / | (102) |
|  | Neonatal rat ventricular CMs | Collagen protected gold nanoparticles, collagen protected silver nanoparticles, collagen hydrogels | Nanoengineered hybrid electroconductive cardiac patches were generated in vitro by combining different nanoparticles with a collagen hydrogel and evaluated in vivo in a mouse model of MI. Only nanogold containing hydrogels were demonstrated to recover cardiac function through neoangiogenesis and increased cell-cell connectivity. | / | (225) |
|  |  |  |  |  |  |
| **CELL ENCAPSULATION** | Adult hBM-MSCs | 3D type 1 rat collagen hydrogels | In vitro generated spheroids of hBM-MSCs and collagen I were useful to model the dynamics of growth factor paracrine effects for stem-cell-based transplantation approaches. | / | (69) |
|  | Mouse BM cells | GelMA cell coating | GelMA coating of mouse BM cells increased the retention of cells in injected infarcted hearts in vivo. A similar effect was observed after challenging these coated cells with decellularized hearts in vitro. Even after GelMA engineering with beta-1-integrin, no cell interaction with H9C2 cells and HUVEC was observed in vitro. Such a retention effect was likely related to collagen binding. | / | (82) |
|  | hES-CMs and hBM-MSCs | Collagen | hBM-MSC incorporation during hES-CMs’ encapsulation in collagen promoted cell survival and maturation in vitro. Moreover, these properties were increased by mechanical loading. | / | (101) |
|  | Rat cardiomyoblasts (H9C2) | Polycaprolactone, calcium peroxide, and GelMA | Oxygen-generating microparticles co-encapsulated with CMs in a gelatin-based hydrogel matrix improved the mechanical properties of the scaffold, as well as cell viability and function in a hypoxic model in vitro. | / | (107) |
|  | Neonatal rat ventricular CMs | Decellularized omentum hydrogel, gold nanoparticles | An ECM‐based hydrogel incorporating gold nanoparticles was generated to encapsulate CMs in vitro. These aggregates were implanted in an ischemia-reperfusion heart rat model by preventing ROS-induced damages on transplanted cells and, thus, the deterioration of cardiac performance. | / | (108) |
|  | hiPS-CMs | Microwells for self-aggregation and gelatin | hiPS-CM spheroids were implanted in female animal models of acute MI (rats) and heart failure (pigs). In both models, they engrafted successfully and were highly vascularized. Arrhythmic events were registered in the porcine model, but no fatal events occurred. | / | (204) |
|  | hiPS-CMs | Microwells for self-aggregation | In a murine MI model, hiPS-CM spheroids resulted in a higher engrafting rate with respect to single cell infusion. | / | (207) |
|  | HUVECs | Alginate, chitosan, and collagen | Encapsulation of HUVEC in alginate/chitosan microcapsules helped to preserve EC viability and function after incorporation in collagen. | / | (224) |
|  |  |  |  |  |  |
| **BIOPRINTING** | ﻿hiPS-CMs, hBM-MSCs, and hECs | GelMA and PEGDA | Four-dimensional cardiac patches in ﻿GelMA and PEGDA at different densities were printed using a beam-scanning stereolithography technique. Co-printed hiPS-CMs ﻿showed high proliferation and beating rate. Functional vascularized cardiac patches were also generated in vitro including hBM-MSCs and hECs in a dynamic culture system. | / | (88) |
|  | Human ventricular CMs and human CFs | GelMA, bovine dermis type I collagen, and G3C1 hydrogel | So-called cMesh and cPatch were generated with 3D-bioprinted hydrogels in GelMA, collagen, and/or G3C1 modifying several physical properties. These hydrogels were used to encapsulate human CMs and CFs with a preserved cell viability in cMesh ones. CMs in cMesh resulted to be interconnected in vitro. Moreover, implantation of tissue engineered cMesh in vivo showed successful engraftment and performance. | / | (89) |
|  | Neonatal rat CMs | Alginate and gelatin | 3D-bioprinted, layered scaffolds of alginate and gelatin were seeded with neonatal rat CMs. Cell attachment, elongation, beating, and response to adrenergic stimulations of these engineered myocardial tissues were suggestive of a functional tissue. | / | (90) |
|  | Rat cardiomyoblasts (H9C2) | Polystyrene and gold nanoparticles | Capillary force lithography was used to generate nanopillars and microwrinkles. Both hierarchical topographies demonstrated to enhance CM maturation. | / | (91) |
|  | hiPS-CMs | PLA | Electric field-driven jet microscale 3D printing allowed to obtain a highly ordered microstructure, which promoted the maturation of hiPS-CMs in vitro. | / | (92) |
|  | Mouse CPCs | PEGDA, Irgacure 819, curcumin, and polyaniline | A conductive ink of PEGDA, Irgacure 819, curcumin, and polyaniline was fabricated by microstereolitography. The scaffold demonstrated to be an optimal microenvironment to support the viability of mouse CPCs in vitro. | / | (93) |
|  | Neonatal rat ventricular CMs | Gold nanowires and alginate | An increased cell viability and alignment was observed in CMs seeded onto scaffolds engineered with gold nanowires. | / | (94) |
|  | Neonatal rat ventricular CMs | Carbon nanotubes, polypyrrole, and PEDOT | The incorporation of conductive polymers in carbon nanotubes led to the generation of a highly biocompatible scaffold able to maintain cell viability and regular beating of CMs. | / | (100) |
|  | hiPS-CMs, hiPS-ECs, and hiPS-SMCs | GelMA | Multiphoton-excited 3D printing was used to generate heart ECM-inspired scaffolds. hiPS- differentiated cells were seeded in vitro into these scaffolds following specific ratios. Derived cardiac patches were transplanted in infarcted murine hearts. Cardiac remodeling and function were ameliorated in treated animals, but cell retention decreased progressively (about 11% at 4 weeks from implantation). | / | (222) |
|  |  |  |  |  |  |
| **ELECTROSPINNING** | CSCs | Electrospun PGA nanofibers and collagen | The collagen sponges incorporating PGA nanofibers increased in vitro the adhesion and proliferation of CSCs, especially in 3D, dynamic settings. | / | (65) |
|  | Neonatal rat CMs and CFs | Electrospun silk engineered with graphene oxide | Electrospun silk fibroin scaffolds engineered with graphene oxide supported the cell viability of co-seeded CMs and CFs in vitro. Once in vivo in MI rats, the scaffolds applied to the epicardial surface increased left ventricular wall thickness, contributed to improve the survival of resident CMs’ and global heart performance. | / | (97) |
|  | Neonatal rat ventricular CMs, human lung fibroblasts, and hiPS | Gelatin, PLGA, and polypyrrole | The electrospun conductive scaffold resulted to be cytocompatible for all cells tested in vitro. | / | (99) |
|  |  |  |  |  |  |
| **SOLUTION BLOW SPINNING** | Rat cardiomyoblasts (H9C2), human CMs, and rat ventricle CMs | PLA and polyurethane, with possible protein modifications (fibronectin, collagen, gelatin, laminin, poly-l-lysine) | Nanofibrous mats of PLA and polyurethane were generated by solution blow spinning and eventually submitted to modification with proteins. They better supported cell viability and orientation of all cell types than simple polystyrene. | / | (95) |
|  |  |  |  |  |  |
| **CELL SHEETS** | Mouse ES-CMs, mouse ES-ECs, and mouse dermal fibroblasts | PIPAAm | Myocardial cell sheets prevascularized with capillary networks were created with CMs and ECs differentiated from ES and dermal fibroblasts. | / | (112) |
|  | ﻿Mouse ES-CMs, mouse neonatal CFs, mouse adult CFs, and mouse adult dermal fibroblasts | PIPAAm | The contraction activity of cardiac cell sheets generated with ES-CMs was sustained only in co-presence of neonatal CFs due to the higher expression of VCAM1. Treatment with soluble VCAM1 improved the functional maturation of ES-CMs. | / | (113) |
|  | Neonatal rat ventricular CMs | PIPAAm, PLGA, and PVA | Cardiac cell sheets were assembled in vitro with a VEGF-releasing electrospun PLGA-PVA mat and implanted in vivo in a subcutaneous rat model. This strategy helped increasing vascularization of cardiac cell sheets. | / | (114) |
|  | hiPS-CMs | PIPAAm | The combination of cardiac cell sheets with the omental flap technique improved ejection fraction in the settings of ischemic cardiomyopathy in a pig model by increasing cell survival with respect to cell sheets alone. | / | (115) |
|  | Bovine carotid artery normal ECs | PIPAAm, poly(butyl methacrylate)-*b*-poly(*N*-isopropylacrylamide), and poly(butyl methacrylate)-*b*-(*N*-acryloylmorpholine) | Micropatterned thermoresponsive materials were generated by microcontact printing and allowed for selective topography cell growth. | / | (116) |
|  | Bovine carotid artery normal ECs | PIPAAm | Thermoresponsive material brush with concentrated cationic surfaces were synthesized. Bovine ECs differently attach to the materials depending on the length of the chains. | / | (117) |
|  | hiPS-CMs | PIPAAm | Tubular cell sheets were obtained by using a demucosalized 5 cm-long, small intestine fragment as vascular bed. | / | (118) |
|  | Mouse ES-CMs, mouse ES-ECs, and mouse ES-SMCs | PIPAAm and gelatin hydrogel microspheres | Gelatin hydrogel microspheres were incorporated into rat cardiac cell sheets in vitro and implanted in a rat model of MI. Engraftment was successful with prompt revascularization and viability maintenance for up to 3 months after implantation. | / | (171) |
|  | hiPS-CMs | PIPAAm and gelatin hydrogel microspheres | Gelatin hydrogel microspheres were incorporated into human cardiac cell sheets in vitro and implanted in a rat model of MI. Functional recovery was sustained for up to 2 months after transplantation. | / | (172) |
|  | Rat dermal fibroblasts, rat endothelial progenitor cells | PIPAAm | Cells sheets were generated in vitro by co-culturing dermal fibroblasts and endothelial progenitor cells. They were implanted in a rat MI model in vivo. A rich vascular network was observed after explant. | / | (173) |
|  | Human amnion or chorion MSCs | Alginate and PDMS | Cell sheets of cardially committed human amnion or chorion MSCs were submitted to mechanical testing. Final modulus, ultimate tensile strength, and maximum strain did not reach the value of the healthy heart muscle at early diastole. | / | (177) |
|  | C-kit-positive CPCs | PIPAAm | C-kit-CPC sheets were implanted in a rat model of MI. Once in vivo, CPCs proliferated, differentiated, and migrated; moreover, the sheets were vascularized. | / | (179) |
|  | hiPS-CMs | PIPAAm | hiPS-CM cardiac cell sheets were transplanted in a nude rat MI model. Implants succesfully engrafted, were vascularized, and contributed to ameliorate host’s heart function. | / | (180) |
|  | Sca1-positive mouse CPCs | PIPAAm | Sca1-CPC sheets were transplanted in a mouse model of MI by attenuating myocardial adverse remodeling through differentiation into functional CMs and paracrine release of VCAM1. | / | (181) |
|  | Neonatal rat CMs | PIPAAm | Cell sheets generated in vitro with rat CMs were implanted in a rat subcutaneous model for up to one year. The grafts were promptly vascularized and developed spontaneous beating activity. | / | (182) |
|  | hiPS-CMs | Not specified | Cell sheets were obtained in vitro by using CMs differentiated from iPS generated from CFs. Transplanted on mouse infarcted hearts for up to 28 days, they engrafted, were vascularized and ameliorated cardiac function. | / | (183) |
|  | CPC-CMs | PIPAAm | In a swine chronic ischemic model, CPC-CM cell sheets integrated with host’s myocardium, increased LV thickness, by improving LVEF and global heart function. | / | (184) |
|  | Rat BM-MSCs | PIPAAm | MSC cell sheets were transplanted on the epicardial surface of rat ischemic hearts. In treated animals, improvement in heart function was observed but no effect on host CM viability was detected. | / | (192) |
|  | Rabbit BM-MSCs | PIPAAm | Autologous BM-MSC cell sheets preconditioned in hypoxic conditions in vitro were transplanted in a senile MI rabbit model. Angiogenesis was accelerated through increased secretion of VEGF and cardiac function improvement was observed. | / | (193) |
|  | Rat AD-MSCs | PIPAAm | AD-MSC cell sheets were transplanted in a rat model of MI. After one month, grafts results to be vascularized and few CMs were observed histologically. Cardiac function was ameliorated in transplanted animals. | / | (195) |
|  | Rat AD-MSCs | PIPAAm | After transplantation in a rat ischemic heart model, AD-MSC sheets improved cardiac remodeling by releasing VEGF, as demonstrated by its abrogation with, irbesartan pretreatment. | / | (196) |
|  | Human umbilical cord MSCs | PIPAAm | Human MSC sheets were transplanted in mouse ischemic hearts, reduced inflammatory state, and preserved hosts’ CMC viability. | / | (199) |
|  | Mouse iPS-CMs | PIPAAm | In a rat model of chronic MI, bioengineered myocardial sheets generated with mouse iPS-CMs reduced adverse cardiac remodeling and ameliorated cardiac function after 4 weeks. | / | (208) |
|  | Human iPS-CMs | PIPAAm | / | First-in-humans, sheets of allogeneic hiPS-CMs were transplanted onto the epicardial surface through a mini-thoracotomy access in a patient affected by ischemic cardiomyopathy. No adverse signs, neither tumorigenicity were detected after 6 months. Wall motion was improved. | (210,211) |
|  |  |  |  |  |  |
| **DECELLULARIZED HEARTS AND EXTRACELLULAR MATRICES** | Rat aortic ECs, neonatal rat ventricular CMs | Decellularized rat hearts | After decellularization, rat hearts were repopulated through the aortic access with neonatal rat CMs and reached a ﻿contractile force around 2.4 mm Hg (2% of adult rat heart function and 25% of 16-week fetal human heart function). | / | (120) |
|  | White leghorn chicken embryonic CMs | Decellularized porcine hearts | Decellularized porcine myocardium was seeded with chicken CMs and supported the formation of sarcomeres, with random orientation. | / | (121) |
|  | Rat BM-MSCs, HUVEC | Decellularized porcine myocardium | Decellularized porcine thick myocardial regions were seeded with rat BM-MSCs or HUVEC. BM-MSCs adhered to the decellularized scaffolds and remained viable. HUVEC created a monolayer on the inner lumen of acellular vessel scaffolding. | / | (122) |
|  | Human BM-MSCs | Decellularized rat hearts | When seeded with human BM-MSCs, decellularized rat heart tissues supported the attachment as well as the migration inside the deeper thickness with high proliferation activity. | / | (123) |
|  | hiPS-derived CPCs | Decellularized mouse hearts | hiPS-derived CPCs seeded into decellularized mouse hearts ﻿migrated, proliferated, and differentiated into CMs, SMCs, and ECs. Moreover, contractive activity was observed. | / | (133) |
|  | Neonatal rat mixed cardiac cells | Decellularized rat hearts | ﻿Through antegrade coronary perfusion, cardiac cells were infused into decellularized hearts and spontaneous contraction started few days after seeding. | / | (138) |
|  | Rat CFs, hES-MSCs | Decellularized CF extracellular matrices | Cell sheets of CFs were decellularized and seeded in vitro with hES-MSCs to evaluate biodistribution in all cardiac organ after implantation of the patch onto the surface of ischemic myocardium in the mouse. | / | (153) |
| Legend: MI: MI; h: human; ES: embryonic stem cells; iPS: induced pluripotent stem cells; CMs: cardiomyocytes; CPCs: cardiac progenitor cells; BM: bone marrow; MSCs: mesenchymal stem cells; ECM: extracellular matrix; CFs: cardiac fibroblasts; PGA: poly(glycolic) acid; 3D: three-dimension; 4D: four-dimension; SMCs: smooth muscle cells; ECs: endothelial cells; HUVECs: human umbilical vein endothelial cells; LVEF: left ventricle ejection fraction; PEGDA: poly(ethylene glycol) diacrylate; PEDOT: poly(3,4-ethylenedioxythiophene); PIPAAm: Poly(N-isoproplyacrylamide; PLA: polylactic acid; PVA: polyvinyl alcohol; PLGA: poly(lactic co-glycolic) acid; PDMS: poly(dimethylsiloxane). | | | | | |

Table 2 Myocardial tissue engineering models of cardiovascular disease

| **CARDIOVASCULAR**  **DISEASE** | **CELL TYPE** | **SCAFFOLD TYPE** | **MAIN TECHNOLOGY** | **KEY**  **RESULTS** | **REF.** |
| --- | --- | --- | --- | --- | --- |
| **MI** | Cardiosphere-derived CPCs and CFs | Decellularized healthy or pathological CFs-synthesized ECM in gel solution | In vitro tissue engineering with hydrogels | Healthy or pathological cardiogels obtained from CFs-synthesized ECM were seeded with CPCs. The interaction with the pathological hydrogel reduced the pro-regenerative paracrine effect of CPCs. | (154) |
|  | hiPS-CMs and CFs | Collagen hydrogels | Heart-on-a-chip | Cardiac engineered tissues were generated with opportune ratio of CMs and CFs. They were exposed to varying oxygen levels. Typical MI sequelae were observed in terms of cytotoxicity, dysregulated contraction, and hypoxic pathways upregulation. | (255) |
|  | hiPS-CMs, hBM-MSCs, and CFs | Hyaluronic acid | 3D bioprinting and spheroids | A scarred myocardial tissue reminiscent of MI remodeling was created by bioprinting and fusing cell spheroids in a spatially oriented manner. Reduction of contractility and arrhythmias were recapitulated in vitro. miRNA therapeutics was tested to counteract progression of adverse remodeling. | (262) |
|  | Rat cardiomyoblasts (H9C2) | GelMA | Single cells and spheroids | H9C2 cells were combined with GelMA alone or after spheroid formation. Seeded hydrogels were submitted to different stiffness regimens. Cardiomyoblasts showed limited cellular adaptation to increasing scaffold stiffness, by overexpressing YAP, MRTF-A, and lamin, and decreasing nuclear size. | (263) |
|  | hiPS-CMs, human ventricular CFs, HUVECs, hAD-MSCs, | Non-adhesive agarose gel | Cardiac organoids and spheroids | Cardiac organoids were generated with specific ratios between myocytes and non-myocytes and stimulated with different oxygen gradients and noradrenaline to simulate early MI events (damage and nervous system compensation). The model was able to recapitulate the cascade of events following ischemic attack in terms of cell death, collagen secretion and altered calcium handling. | (264) |
|  | Rat neonatal CMs, hiPS-ECs or HUVECs | RGD-conjugated PEGDA | Cell encapsulation | Oxidative stress was applied to 3D capsules of CMs and ECs. HIF-1-alpha signaling was investigated also by knock in strategy, by revealing the essential role of ECs in cardioprotection. | (265) |
|  | Rat neonatal CMs, hiPS-ECs | RGD-conjugated 4-arm PEG-ACRL | Cell encapsulation | With a similar approach to Acun et al., the group further investigated the EC cardioprotective effects and highlighted the involvement of mitochondrial gene pathways and adrenergic signaling. | (266) |
|  | hiPS-CMs, hCPCs | Polystyrene for cell aggregation | Cell aggregation, tank bioreactor | CM aggregates were submitted in stirred tank bioreactors to changing oxygen gradients. They replied by reducing cell viability and undergoing cell disarray and modifying their secretome towards pro-angiogenesis and pro-inflammation. | (267) |
|  | Mouse cardiac cells, hiPS-CMs, hCFs, human coronary artery ECs | / | Cardiac spheroids | Cardiac spheroids were generated by opportune mix of myocytes and non-myocytes. They were submitted to different hypoxic conditions. The model allowed to confirm the lower resistance of CMs to ischemia than ECs and CFs. | (268,269) |
|  | hBM-MSCs, rat neonatal CMs | RGD-modified alginate | Cell encapsulation | hBM-MSCs and rat CMs were encapsulated in RGD-modified alginate and exposed to H_2_O_2_-enriched microenvironment. The presence of hBM-MSCs contributed to maintain higher the survival rate, as well as the pro-regeneration properties of CMs although the hypoxic settings. | (270) |
|  | Rat BM-MSCs | Enriched PEGDA | In vitro tissue engineering with hydrogels | Different rigidity conditions were obtained by varying the percentage of PEGDA to mimic the MI scar environment. Seeded BM-MSCs drastically reduced their pro-regenerative paracrine abilities. These outcomes were useful to get more insights on the unmet promises of BM-MSCs infused in MI patients’ clinical trials. | (271) |
|  |  |  |  |  |  |
| **CARDIAC**  **FIBROSIS** | hCFs, hiPS-ECs | Fibrin | Heart-on-a-chip | 3D vascular models were exposed to two TGF-beta regimens. Alterations in microvasculature, permeability, and extravascular diffusivity were observed recapitulating the typical signs of cardiac fibrosis. | (272) |
|  | Human fetal CFs and hiPS-CMs | GelMA | In vitro tissue engineering with hydrogels | A myocardial model was generated by combining human fetal CFs, hiPS-CMs, and GelMA. After conditioning with TGF beta 1, higher levels of alpha smooth muscle actin, periostin, collagen I, and metalloproteinase 2 were detected at the molecular and protein levels. The administration of the anti-fibrotic drug pirfenidone reverted only partially this pathological phenotype. | (273) |
|  | Human cardiosphere-derived stromal cells | GelMA | In vitro tissue engineering with hydrogels | In a cardiac fibrosis model generated with human cardiospheres-derived cells and GelMA at varying stiffness degrees, TGF beta 1 stimulation overexpressed YAP and induced fibrosis. The administration of the YAP antagonist Verteporfin halted fibrosis progression, proposing the use of this drug besides its FDA-approved application in age-related macular degeneration. | (38) |
|  | hCFs | Polycaprolactone, dopamine coating, and gelatin | In vitro tissue engineering with synthetic scaffolds | 2D and 3D myocardial engineered models of early MI fibrotic lesion were generated by combining hCFs, polycaprolactone, and mussel-inspired dopamine-coated surface gelatin grafting. The scaffolding reminiscent of the scar ECM was able to condition CFs to myofibroblasts, as characteristically observed in cardiac fibrosis. Atrial and ventricular CFs were both used demonstrating the possibility to generate atrial and ventricular models of cardiac fibrosis. | (275) |
|  | Neonatal rat CFs | Polyacrylamide | In vitro tissue engineering with hydrogels | Three cardiac fibrosis models were generated seeding CFs in differently stiff polyacrylamide gels. The administration of candesartan reduced fibrotic responses in CFs and, in particular, had strong effects on the expression of FAK gene and its downstream targets. Through several inhibition experiments, Zhu et al. were able to identify FAK as a key target for possible pharmacological treatments. | (277) |
|  | Healthy and pathological hCFs | Collagen | In vitro tissue engineering with hydrogels | Cardiac fibrosis models were obtained by seeding CFs in a collagen mixture at different stiffness degrees or in presence of fibrotic signals. By substituting healthy CFs with analogous cells derived from patients with ischemic or dilated cardiomyopathy, the in vitro progression of cardiac fibrosis was accelerated in the case of cells derived from hypertensive patients. | (278) |
|  | Rat CFs | Collagen, alginate, gelatin, and PEGDA | 3D bioprinting, cell encapsulation | A drug testing cardiac fibrosis platform was manufactured through a plant-bioinspired approach (catcher-like scaffold microstructures), rat CFs, and TGF beta 1. | (279) |
|  | Rat cardiomyoblasts (H9C2) and human dermal fibroblasts | Fibrin | Heart-on-a-chip | Cardiac tissue chips were obtained by seeding H9C2 cells and dermal fibroblasts in a fibrin hydrogel. Pressure overload was imposed, and myofibroblast switch was observed for dermal fibroblasts, as well as cell responses related to inflammation and oxidative stress. | (280) |
|  |  |  |  |  |  |
| **CARDIAC HYPERTROPHY** | Rat neonatal CMs and CFs | Collagen gels | Pneumatic microfluidic microbioreactor | Cardiac microtissues were generated by seeding CMs and CFs in collagen gels. The application of an increasing strain induced overexpression of atrial natriuretic peptide, myosin heavy chain, and actin, the genes of the so-called fetal cardiac program, physiologically switched off in adult life. | (254) |
|  | hCFs, hES- and hiPS-CMs | Collagen and Matrigel | Heart-on-a-chip | Atrial or ventricular disease microchip models were developed with a hydrogel of collagen and Matrigel, human CFs and chamber-specific CMs. Left ventricular hypertrophy with the typical molecular signature was recapitulated by using primary hypertension patient-specific cells instead of those derived from healthy subjects. | (244) |
|  | hiPS-CMs | Fibronectin-decorated elastomer | Micro-heart muscle arrays | Microengineered tissues with higher elastomer stiffness showed an increased cell size, beating frequency, higher calcium influx, and a reduced traction force. | (281) |
|  |  |  |  |  |  |
| **CARDIOTOXICITY** | hiPS-CMs and hiPS- fibroblasts | Silicon rubber | Micro-heart muscle arrays | Micro-heart muscles showed reproducible and physiological responses to verapamil and inotropic reply to isoproterenol. | (287) |
|  | hiPS-CMs and hiPS-mesenchymal cells | PIPAAm | Cell sheets | Cell sheets of CMs alone or in combination with non-CM cells were generated with gelatin-coated PIPAAm. E-4031 administration induced tachyarrhythmia with spiral wave reentry waveforms as in patients affected by torsade de pointes. | (288) |
|  | hiPS-CMs | Decellularized porcine myocardium hydrogel and reduced graphene oxide | In vitro tissue engineering with hydrogels, electroconductive materials | Exposure of myocardial engineered tissues, composed of decellularized porcine myocardium, graphene oxide, and hiPS-CMs, to cisapride provoked the prolongation of action potential and abnormalities in the beating frequency. These signs resemble those observed during fatal arrhythmias and long QT provoked by cisapride in patients submitted to gastrokinetic treatment. | (290) |
|  | hiPS-CMs and CFs | Non-adhesive agarose | Cardiac spheroids | Cardiac spheroids were generated by mixing hiPS-CMs and CFs. Exposure to the pollutant bisphenol activated a complex response. In silico modeling integrating experimental results and clinical observations identified a two-step reply to the pollutant, given by immediate potassium channel blockade and shortening of the action potential duration. | (291) |
|  | hiPS-CMs, human cardiac microvascular ECs, and hCFs | Ultra-low adhesion polystyrene | Cardiac spheroids | ﻿29 FDA-approved cardiotoxins (14 structural and 15 non- structural) were tested and induced rhythm alterations, by acting on distinctive pathways related to cell viability and cardiac tissue metabolism. | (292) |
|  | hiPS-CMs, human ventricular CFs, HUVECs, hAD-MSCs, | Non-adhesive agarose gel | Cardiac organoids and spheroids | Cardiac organoids were generated with specific ratio between myocytes and non-myocytes and stimulated with different oxygen gradients and doxorubicin to simulate the myocardial response in patients affected by MI and undergoing chemotherapy. This unprecedented investigation recapitulated the typical cardiac tissue signs observed in these patients and might be useful to identify personalized dosage treatments or novel targets. | (264) |
|  | HCMs (AC16 cell line) | Gelatin hydrogel | 3D bioprinting and cardiac spheroids | Doxorubicin-induced cardiotoxicity was found to be antagonized in a 3D bioprinted, cardiac spheroid system by administering N-acetylcysteine and Tiron able to inhibit caspase 3 apoptotic pathway. | (294) |
| Legend: MI: myocardial infarction; h: human; CPCs: cardiac progenitor cells; CFs: cardiac fibroblasts; ECM: extracellular matrix; BM: bone marrow; MSCs: mesenchymal stem cells; ECs: endothelial cells; HUVECs: human umbilical vein endothelial cells; AD: adipose tissue-derived; RGD: arginine-glycine-aspartic acid; PEG-ACRL: Acrylate- poly(ethylene glycol); PEGDA: poly(ethylene glycol) diacrylate. | | | | | |
